# The Structural Basis of ATP as an Allosteric Modulator

**DOI:** 10.1371/journal.pcbi.1003831

**Published:** 2014-09-11

**Authors:** Shaoyong Lu, Wenkang Huang, Qi Wang, Qiancheng Shen, Shuai Li, Ruth Nussinov, Jian Zhang

**Affiliations:** 1 Department of Pathophysiology, Key Laboratory of Cell Differentiation and Apoptosis of Chinese Ministry of Education, Shanghai JiaoTong University, School of Medicine, Shanghai, China; 2 Cancer and Inflammation Program, Leidos Biomedical Research, Inc., Frederick National Laboratory, National Cancer Institute, Frederick, Maryland, United States of America; 3 Department of Human Genetics and Molecular Medicine, Sackler School of Medicine, Sackler Institute of Molecular Medicine, Tel Aviv University, Tel Aviv, Israel; 4 Shanghai Key Laboratory of Tumor Microenvironment and Inflammation, Shanghai Jiao Tong University School of Medicine, Shanghai, China; Fudan University, China

## Abstract

Adenosine-5’-triphosphate (ATP) is generally regarded as a substrate for energy currency and protein modification. Recent findings uncovered the allosteric function of ATP in cellular signal transduction but little is understood about this critical behavior of ATP. Through extensive analysis of ATP in solution and proteins, we found that the free ATP can exist in the compact and extended conformations in solution, and the two different conformational characteristics may be responsible for ATP to exert distinct biological functions: ATP molecules adopt both compact and extended conformations in the allosteric binding sites but conserve extended conformations in the substrate binding sites. Nudged elastic band simulations unveiled the distinct dynamic processes of ATP binding to the corresponding allosteric and substrate binding sites of uridine monophosphate kinase, and suggested that in solution ATP preferentially binds to the substrate binding sites of proteins. When the ATP molecules occupy the allosteric binding sites, the allosteric trigger from ATP to fuel allosteric communication between allosteric and functional sites is stemmed mainly from the triphosphate part of ATP, with a small number from the adenine part of ATP. Taken together, our results provide overall understanding of ATP allosteric functions responsible for regulation in biological systems.

## Author Summary

The endogenous ATP can be regarded as a substrate and an allosteric modulator in cellular signal transduction. We analyzed the properties of allosteric and substrate ATP-binding sites and found that the allosteric ATP-binding sites are less conserved than the substrate ATP-binding sites. Allosteric ATP molecules adopt both compact and extended conformations in the allosteric binding sites, while substrate ATP molecules adopt extended conformations in the substrate binding sites. The two different conformational characteristics may be responsible for ATP to exert distinct biological functions in cell signaling. In addition, to our knowledge, this study illuminates the first comprehensive atomic level investigations of ATP access to the corresponding allosteric and substrate ATP-binding sites. Specially, both the adenine and triphosphate parts of ATP could be an allosteric trigger to propagate the signal from the allosteric to functional sites. The detailed mechanism presented in this study may apply to other enzymes in complex with allosteric or substrate ATP molecules, and provide important insights for the molecular basis of ATP acting as a substrate and an allosteric modulator in cell signaling.

## Introduction

Adenosine-5’-triphosphate (ATP), the naturally occurring nucleotide used in cells as a coenzyme, serves as the “energy currency of the cell” in intracellular energy metabolism [1]. In eukaryotes, the majority of ATP is manufactured in the chloroplasts (for plants) or in the mitochondria (for both plants and animals) as a result of cellular processes including fermentation, respiration and photosynthesis [2], [3]. The well-known functional role of ATP in cells is the hydrolysis of its high-energy phosphate bond to provide energy for biological processes. In addition, intracellular protein modification via ATP as a substrate, known as phosphorylation, orchestrates a fine-tuned network for the cell to switch on diverse signal transduction processes, such as proliferation, gene transcription, metabolism, kinase cascade activation, and membrane transport [4].

Although ATP is one of the most productive endogenous ligands in cells, striking evidence from clinical practice demonstrates that intravenous or oral administration of ATP may have pivotal effects on the treatment of pathological processes, including the regulation of diverse processes (coronary blood flow [5]–[7], neurotransmission [8], muscle contraction [9], and inflammation [10], [11]), the protection of the myocardium and the maintenance of the homeostasis of the cardiovascular system [12], [13]. The usage of ATP indicates that ATP could play even more roles in physiological/pathological regulation.

Recently, the determination of allosteric ATP-binding sites provided new evidences that ATP can act as an allosteric modulator regulating protein function [14], [15]. For instance, in the mismatch repair initiation protein MutS, ATP binding to the nucleotide-binding site triggers long-range interactions between the ATPase and DNA-binding domains [16]. In the chaperonin GroEL, binding of ATP to a unique pocket in the equatorial domains of GroEL allosterically initiates a series of conformational changes triggering the association of the cochaperonin GroES [17]. As a extracellular messager, ATP was confirmed to allosterically involve in activation mechanism of transmembrane protein P2X receptors [18], [19].

The role of ATP in cell signaling is known to serve not only as a substrate but also as an allosteric modulator. However, little is understood about this critical behavior of ATP in allosteric binding sites. The dynamic process of ATP access to the allosteric and substrate binding sites and the allosteric mechanism for ATP molecules are also unclear. Here, we addressed these questions by combining bioinformatic analyses, molecular dynamics (MD) and nudged elastic band (NEB) simulations. The results suggest that allosteric ATP-binding sites have evolved along different evolutionary pathways compared to the extremely conserved substrate ATP-binding sites. ATP molecules adopt both compact and extended conformations in the allosteric binding sites but conserves extended conformations in the substrate binding sites, and both compact and extended conformations of ATP are dominant in solution. We follow the preference of ATP access to the allosteric and substrate binding sites at atomic resolution and explore the mechanism of ATP-triggered allostery. Understanding the regulatory mechanism of ATP in biological systems as a substrate and an allosteric modulator in cell signaling is of fundamental importance.

## Results

**Figure 1:**
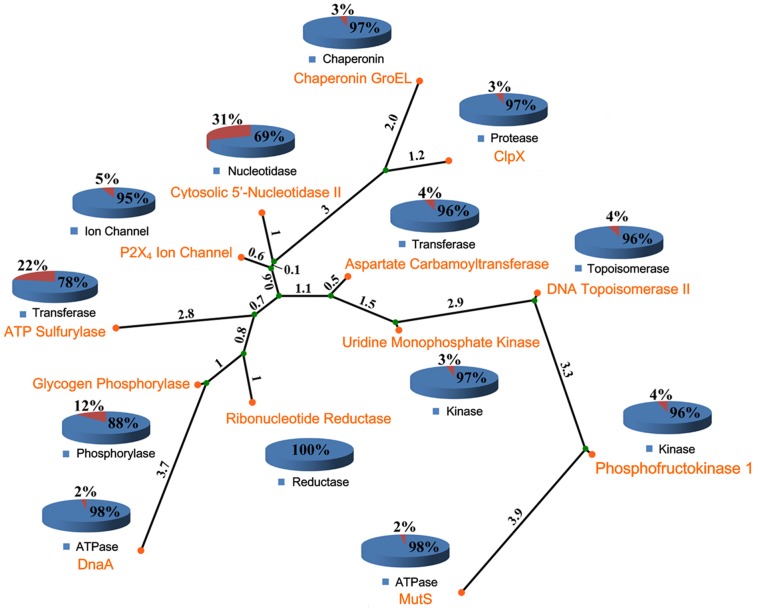
Phylogenetic tree of 13 allosteric proteins. The phylogenetic tree was derived using ClustalX program. The tree structure consists of nodes (represented as circles) and branches (the connecting lines). The internal and external nodes are represented by green and orange circles, respectively. Phylogenetic distance is proportional to branch length. The allosteric proteins belonging to different protein families are depicted in the pie chart. Orange represents the percentage of the number of proteins with a sequence identity greater than 30% when compared to the reported allosteric protein in this protein family deposited in ASD.

Thirteen proteins that are allosterically modulated by ATP, constituting the ATP allosteric dataset (Table 1), were selected from the Allosteric Database (ASD v2.0) [15] (see Materials and Methods). As enumerated in Tables S1 and S2, the sequence identities and the structural differences (root-mean-square deviation, RMSD) between these allosteric proteins are predominately below 30% and larger than 20 Å, respectively. The phylogenetic tree constructed for the 13 allosteric proteins displayed a wide distribution of these allosteric proteins (Figure 1). These data indicated their low redundancy. The ATP substrate dataset (Table S3), composed of 24 co-crystal structures of protein-ATP complexes that exhibit structural diversity, was derived from the Protein Database Bank (PDB) database [20].

**Table 1:** Thirteen proteins modulated by allosteric ATP.

Protein name	Organism		PDB entry	Resolution (Å)	Ref.
Glycogen phosphorylase	Homo sapiens		1FA9	2.40	34
ATP sulfurylase	Penicillium chrysogenum		1I2D	2.81	43
Chaperonin GroEL	Escherichia coli		1KP8	2.00	17
Phosphofructokinase 1	Escherichia coli		1PFK	2.40	44
MutS	Escherichia coli		1W7A	2.27	16
DnaA	Aquifex aeolicus		2HCB	3.51	41
Uridine monophosphate kinase	Bacillus anthracis		2JJX	2.82	22
Cytosolic 5’-nucleotidase II	Homo sapiens		2XCW	1.90	73
ClpX	Escherichia coli		3HWS	3.25	45
Ribonucleotide reductase	Escherichia coli		3R1R	3.00	74
Aspartate carbamoyltransferase	Escherichia coli		4AT1	2.60	75
P2X_4_ ion channel	Danio rerio		4DW1	2.80	19
DNA Topoisomerase II	Saccharomyces cerevisiae		4GFH	4.41	42

### Allosteric ATP-binding sites are less conserved than substrate ATP-binding sites

**Figure 2:**
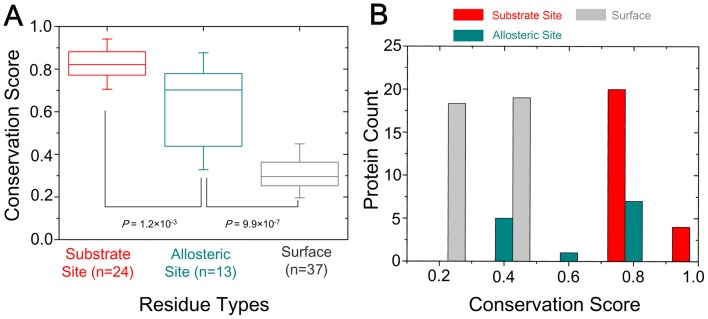
Comparison of evolutionary conservation of allosteric and substrate ATP-binding sites. (A) Distributions of conservation scores for residues in the allosteric and substrate ATP-binding sites as well as in the surface of proteins. (B) Distributions of average conservation scores for residues in the substrate and allosteric ATP-binding sites and the surface per protein. The statistical significant (*P*-value) was calculated by the Mann-Whitney U test.

To elucidate differences in the evolutionary characteristics of allosteric and substrate ATP-binding sites, we systematically explored the sequence conservation of ATP-binding sites in the ATP allosteric and substrate datasets. Sequence conservation scores for residues in the allosteric and substrate ATP-binding sites as well as in the surface of proteins were assessed by means of multiple sequence alignments of homologous sequences (see Materials and Methods for details). As shown in Figure 2A, residues in the allosteric ATP-binding sites, with the average conservation score of 0.63, were less conserved compared to residues in the substrate ATP-binding sites, with the average conservation score of 0.83 (*P* = 1.2×10^−3^). Yet, the residues in the allosteric and substrate ATP-binding sites are markedly more conserved compared to residues in the rest of the surface; the average conservation score of residues in the latter is 0.31 (*P* = 9.9×10^−7^). Furthermore, the average conservation score distributions of residues in the allosteric and substrate ATP-binding sites as well as in the surface for each protein sequence were analyzed. As shown in Figure 2B, remarkably, the average conservation score of residues in the allosteric ATP-binding sites show a wide distribution in a range from 0.4 to 0.8. These results are consistent with a previous study, as done by Yang *et al.* [21], that allosteric sites are evolutionarily variable against catalytic sites. Taken together, these data indicate that allosteric ATP-binding sites have evolved along different evolutionary pathways compared to the conserved substrate ATP-binding sites.

### Pre-existence of the compact and extended conformations of ATP in solution

**Figure 3:**
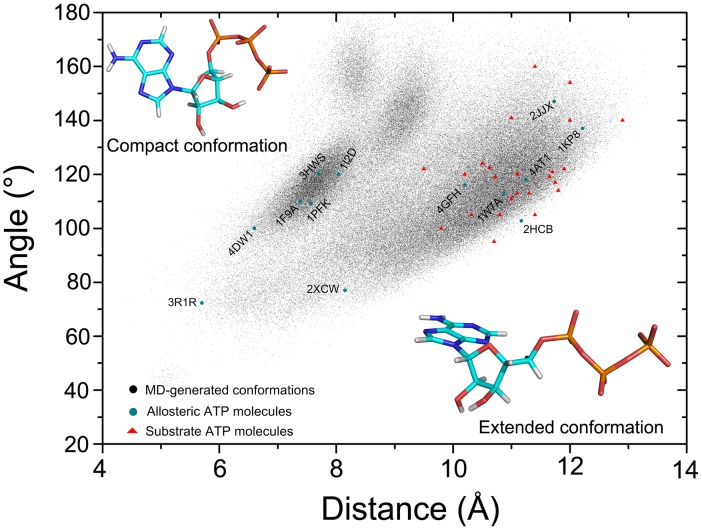
Comparison of conformational preferences of allosteric and substrate ATP molecules. The landscapes with the reaction coordinates of distance (defined by the distance from the P_γ_ atom to the centroid of adenine moiety) and angle (defined by the centroids of triphosphate, ribose and adenine moieties) were plotted on ATP conformations from the MD trajectory. The compact and extended structures of ATP correspond to the two major conformation regions. The bound ATP molecules in the allosteric and substrate datasets were mapped to the MD-generated ATP conformations.

Given the significant structural differences between the allosteric and substrate ATP-binding sites that may accommodate diverse ATP conformations, we first simulated the ATP in solution to explore its conformational preferences in the unbound state. The landscapes with the reaction coordinates of distance (defined by the distance from the P_γ_ atom to the centroid of adenine moiety) and angle (defined by the centroids of triphosphate, ribose and adenine moieties) were calculated on ATP conformations (200,000 snapshots) from the MD trajectory. As shown in Figure 3, two major conformation regions exist in solution, one region with distance values from 6.5 Å to 8.5 Å and angles between 100° and 130°, the other with distance values from 9.3 Å to 12.8 Å and angles ranging between 90° and 140°. An analysis of the average structures of ATP corresponding to the two conformation regions indicates that the former region represents the compact conformation of ATP, whereas the latter represents the extended conformation. These data suggest the pre-existence of the compact and extended conformations of ATP in solution.

### Allosteric ATP molecules adopt both compact and extended conformations, whereas substrate ATP molecules adopt extended conformations in proteins

Next, we investigated the conformational properties of ATP molecules in the allosteric and substrate binding sites by comparing the mean structural differences (RMSD) between each ATP molecule and the remaining ATP molecules in the ATP allosteric and substrate datasets. As shown in Figure S1, the average RMSD value for the 13 allosteric ATP molecules is 1.83 Å. By contrast, the average RMSD value for the 24 substrate ATP molecules is 1.37 Å (*P* = 7.1×10^−5^), revealing smaller conformational variations of ATP in the substrate binding sites. This distinction could be because of the differences in the evolutionary conservation of the allosteric and substrate ATP-binding sites. To further elucidate the structural propensities of ATP molecules in the allosteric and substrate binding sites, we mapped the bound ATP molecules in the allosteric and substrate datasets to the MD-generated ATP conformations. As shown in Figure 3, ATP molecules in the allosteric binding sites show a wide distribution with the distance values ranging from 5.7 Å to 12.2 Å. Inspection of these ATP molecules reveals that seven cases adopt compact conformations (PDB: 1FA9, 1I2D, 1PFK, 3HWS, 2XCW, 3R1R, and 4DW1), while the remaining six cases adopt the extended conformations (PDB: 1W7A, 4AT1, 4GFH, 2HCB, 2JJX, and 1KP8). These data suggest that allosteric ATP molecules can adopt both the compact and extended conformations in the allosteric binding sites of proteins. However, as for the substrate ATP molecules, the analysis reveals that they adopt the extended conformations in the substrate binding sites of proteins.

### ATP molecules access the allosteric and substrate binding sites

The functions of ATP *in vivo* are triggered by the protein transitioning from the free unbound state to the bound state. The uridine monophosphate (UMP) kinase of *Bacillus anthracis* was chosen as a good model to investigate the dynamic process of ATP binding. This kinase not only binds ATP as a substrate in its active site but also has allosteric ATP-binding sites near the center of the hexameric structure [22]. This property of the kinase permits exploration of which site ATP preferentially binds to in solution. The UMP kinase catalyzes the transfer of the γ-phosphoryl group from ATP to UMP in the active site, which is essential for cellular growth in bacteria; the catalytic reaction is regulated by ATP in the allosteric site.

We first examined the sequence conservation of residues in the allosteric and substrate ATP-binding sites of UMP kinase family. As shown in Figure S2 and Table S4, allosteric ATP-binding sites (average conservation score = 0.44) are significantly less conserved compared to substrate ATP-binding sites (average conservation score = 0.77) (*P* = 6.1×10^−4^), but more conserved than the rest of surface residues (average conservation score = 0.25) (*P* = 2.2×10^−3^). The structural characteristics of ATP binding at the allosteric and active sites of UMP kinase were then explored using MD simulations.

#### Structural characteristics of ATP in the allosteric sites of UMP kinase

**Figure 4:**
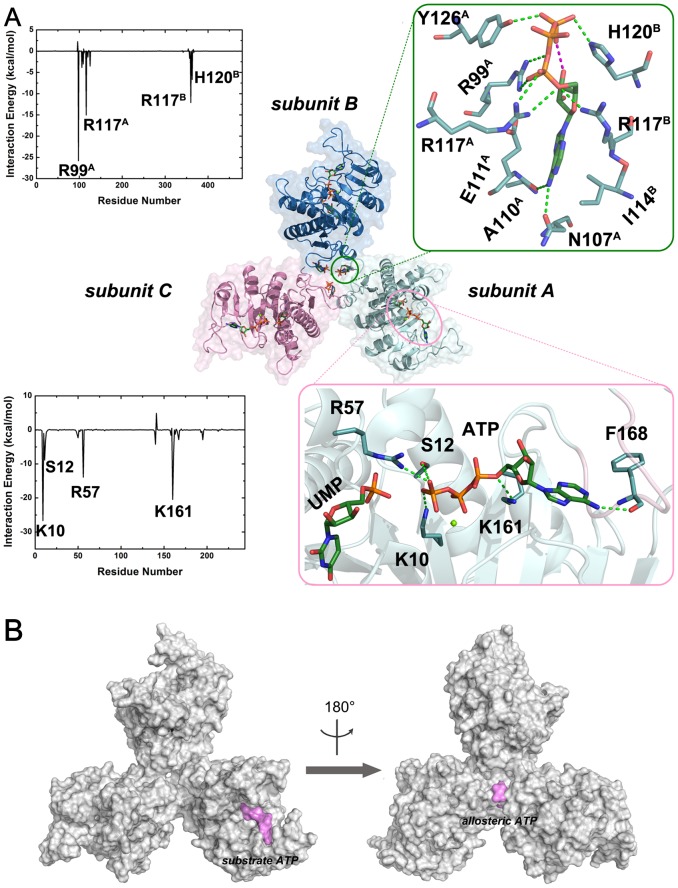
The interactions between UMP kinase and allosteric and substrate ATP molecules. (A) The energy contributions to the binding of allosteric and substrate ATP molecules to the UMP kinase, and the binding modes between the allosteric and substrate ATP molecules and the UMP kinase. The green dashes represent hydrogen bonds. The hydrogen atoms are not displayed for clarity. (B) The volume and shape of the allosteric and substrate ATP-binding sites of the UMP kinase.

In the allosteric ATP-binding sites, three identical ATP molecules were bound at the interfaces between neighboring subunits in the homotrimeric structure of the UMP kinase [22], and their conformational behavior was very similar in the MD simulations. Therefore, we primarily focused on the allosteric ATP molecule at the interfaces between subunits A and B of the UMP kinase (Figure 4).

The analysis of interaction energies between UMP kinase and ATP every 10 ns interval trajectories revealed that the interaction energies converged after 50 ns MD simulations (Figure S3). To elucidate the critical residues for binding the allosteric ATP molecule, the decomposition of interaction energies between each residue in subunits A and B of the UMP kinase and ATP were averaged over the simulation of the ATP-bound state in the last 10 ns MD trajectories. As shown in Figure 4A, major favorable energy contributions come from Arg99, Asn107, Ala110, Arg117 and Tyr126 in subunit A of the UMP kinase and Arg117 and His120 in subunit B. The stabilization energies stem mainly from the positively charged residues Arg99 (A), Arg117 (A, B) and His120 (B). These indicate that the electrostatic interactions are primarily responsible for the binding of the allosteric ATP molecule. To further uncover its binding mode, the most representative structure was extracted from the MD trajectory using a cluster analysis. As shown in Figure 4A, three basic residues, Arg99 (A), Arg117 (A, B) and His120 (B), protrude to interact with the negatively charged phosphate groups. The γ-phosphate group is hydrogen-bonded to the side chain of Tyr126 (A), whereas the guanine moiety interacts through bifurcated hydrogen bonds with backbone atoms of Asn107 (A) and Ala110 (A). The guanine ring is sandwiched by two hydrophobic side chains Glu111 (A) and Ile114 (B). The most prominent feature is the formation of an internal hydrogen bond involving an oxygen atom of the γ-phosphate group and the hydroxyl group of the ribose.

#### Structural characteristics of ATP in the substrate sites of the UMP kinase

For the substrate binding site, ATP was probed at subunit A of the UMP kinase. As shown in Figure 4A, an analysis of the interaction energy between ATP and UMP reveals that the major favorable energy contributions originate from Lys10, Ser12, Arg57 and Lys161. To obtain an insight into the binding mode of the substrate ATP molecule, the most representative structure was extracted from the MD trajectory cluster analysis. As shown in Figure 4A, residues Lys10, Ser12, Arg57 and Lys161 engage in electrostatic or hydrogen bonding interactions with the negatively charged phosphate groups. The guanine ring is encapsulated by the highly conserved motif 165-DGVFTSDP-172 of the UMP kinase. In the substrate binding site, the polyphosphate group of the ATP molecule points to the phosphate group of UMP in an end-to-end alignment. In this optimal organization, a direct nucleophilic attack by the terminal oxygen atom of UMP on the γ-phosphate group of ATP occurs. This is consistent with other crystal structures of enzymes, such as the cAMP-dependent protein kinase A [23], cyclin-dependent kinase 2 [24], and insulin receptor tyrosine kinase [25], determined in complex with ATP and substrates.

#### NEB reveals the main pathway for the binding of an allosteric ATP molecule

**Figure 5:**
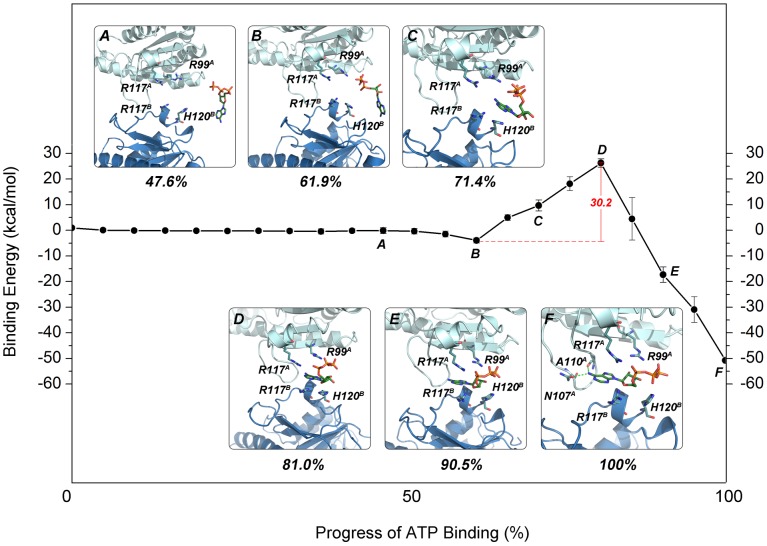
The energy landscape of ATP binding to the allosteric ATP-binding site of the UMP kinase as a function of the progress of ATP binding. (A) The six snapshots of the allosteric ATP binding pathway represent the following configurations: A, the unbound state; B, the “encounter complex”; C, an intermediate state featuring unfavorable electrostatic repulsions between Arg117^A,B^ and adenine; D, the relatively high-energy state; E; an intermediate state featuring favorable electrostatic interactions between the UMP kinase and ATP; F, the fully bound state. The ATP and its interacting residues are shown as sticks, whereas subunits A and B of the UMP kinase are shown in pale cyan and light blue, respectively. The green dashes represent hydrogen bonds. The hydrogen atoms are not displayed for clarity. The error bars represent standard deviations of binding energies for the 20 snapshots from 20 trajectories.

The pathway ATP takes from the solution to the allosteric binding site in the UMP kinase was investigated by twenty nudged elastic band (NEB) simulations from different initial states of ATP in solution. The energy landscape for the pathway was calculated using the MM/PBSA [26], [27] approach based on twenty-two images taken from each NEB trajectory. The dynamic behavior of the ATP binding process in the twenty simulations converged. Thus, one of the twenty trajectories was selected to probe the binding process. Upon examination of the representative snapshots, the process for the binding of allosteric ATP molecule was described by six states which are characterized in three main stages (Figure 5).

The first stage is relevant to the binding of the triphosphate moiety of ATP and the formation of an encounter complex. Figure 5A represents the unbound state, in which no direct contact between ATP and the UMP kinase occurs. Figure 5B manifests the encounter complex between ATP and the UMP kinase in which the ATP triphosphate moiety interacts with the positively charged residue Arg99 (A) of the UMP kinase. The binding is a favorable process given the electrostatic attraction.

The second stage is associated with the binding of the nucleoside moiety of ATP and the formation of an intermediate structure. As shown in Figure 5C, as the triphosphate moiety engages in the interactions with Arg99 (A), the adenine base flips inward and participates in an aromatic-aromatic interaction with His120 (B) in the allosteric ATP-binding site. However, because of the rollover of the adenine base, unfavorable electrostatic repulsions occur between the amino group of the adenine ring and the positively charged residue Arg117 (A, B). With further binding of ATP, as shown in Figure 5D, the aromatic-aromatic interaction between the adenine moiety and His120 (B) is abrogated, and the electrostatic repulsions strengthen between the amino group of the adenine moiety and Arg117 (A, B), which results in the highest energy state corresponding to the intermediate structure. In this process, the energy difference between the highest (state D) and lowest energy (state B) states of binding is 30.2 kcal/mol (the energy barrier of each NEB simulation was listed in Table S5).

The third stage involves further binding of the nucleoside moiety of ATP and the formation of a product structure. With the movement of ATP inward into the allosteric site, as shown in Figure 5E, Arg99 (A) and Arg117 (A) partake in favorable electrostatic interactions with the triphosphate moiety of ATP, and the unfavorable electrostatic repulsions between the amino group of the adenine moiety and Arg117 (A, B) is abolished. As shown in Figure 5F, further movement of ATP enables the amino group of the adenine moiety to form two hydrogen bonds with Asn107 (A) and Ala110 (A), respectively. This hydrogen bonding is coupled with the basic residues Arg99 (A), Arg117 (A, B) and His120 (B) protruding to interact with the negatively charged triphosphate moiety of ATP, resulting in the lowest energy state corresponding to the product structure.

#### NEB reveals the main pathway for the binding of the substrate ATP molecule

**Figure 6:**
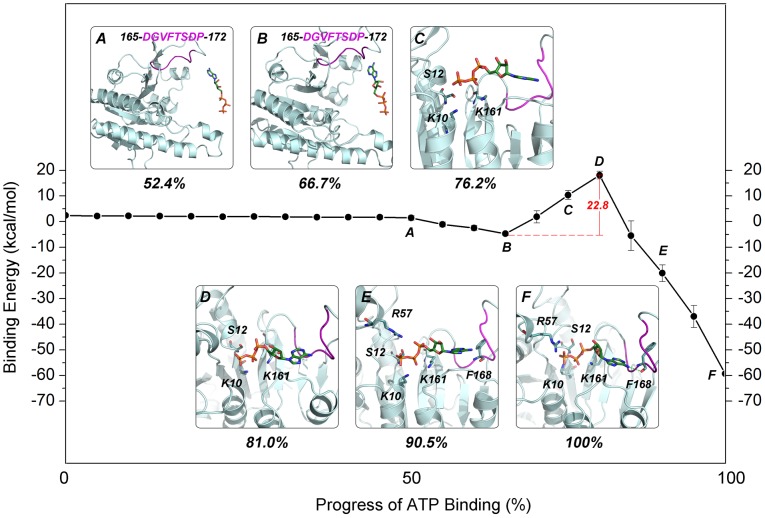
The energy landscape of ATP binding to the substrate ATP-binding site of the UMP kinase as a function of the progress of ATP binding. (A) The six snapshots of the substrate ATP-binding pathway represent the following configurations: A, the unbound state; B, the “encounter complex”; C, an intermediate state in which ATP does not interact with Lys10, Ser12 and Lys161; D, the relatively high-energy state; E; an intermediate state in which ATP interacts with Lys10, Ser12, Arg57, and Lys161; F, the fully bound state. The 165-DGVFTSDP-172 motif of UMP kinase in the recognition of the adenine moiety of ATP is shown in magenta. The ATP and its interacting residues are shown as sticks, whereas subunit A of the UMP kinase is shown in pale cyan. The green dashes represent hydrogen bonds. The hydrogen atoms are not displayed for clarity. The error bars represent standard deviations of binding energies for the 20 snapshots from 20 trajectories.

Figure 6 shows the binding energy between ATP and the UMP kinase during the binding of the substrate ATP molecule to subunit A of the UMP kinase. This profile unveils the atomic details of the binding of the substrate ATP. Inspecting the representative structures along the binding path shows that the process can be described by six states which are also characterized by three main stages.

The first stage encompasses the binding of the adenine moiety of ATP and the formation of the encounter complex. Figure 6A depicts the unbound state in which ATP does not interact directly with the UMP kinase. Figure 6B shows the encounter complex between ATP and the UMP kinase in which the 165-DGVFTSDP-172 motif of UMP kinase is involved in the recognition of the adenine moiety of ATP. This conserved motif has been determined in bacterial UMP kinases, and the related aspartate and glutamate kinases also specifically recognize the adenosine nucleobase [28], [29].

In the second stage, the triphosphate moiety of ATP follows the encounter complex and is involved in an intermediate structure. With the adenine moiety of ATP encased by the 165-DGVFTSDP-172 motif of the UMP kinase, the triphosphate moiety moves into the active site (Figure 6C). With further movement of the triphosphate moiety, it participates in polar interactions with Lys10, Ser12 and Lys161. In this process, the loss of the desolvation energy of ATP is larger than the van der Waals and electrostatic contributions from the interactions between ATP and the UMP kinase. This interaction produces an unfavorable binding process and yields the highest energy state corresponding to the intermediate structure (Figure 6D). In this process, the energy difference between the highest (state D) and lowest energy (state B) states of binding is 22.8 kcal/mol (the energy barrier of each NEB simulation was listed in Table S5).

The third stage is pertinent to the further binding of the triphosphate and adenine moieties of ATP (Figure 6E) and the formation of the product structure. As shown in Figure 6F, the complete binding of ATP enables the amino group of the adenine moiety to interact with the backbone carbonyl group of Phe168 via hydrogen bonding, and the triphosphate moiety displays polar interactions with Lys10, Ser12, Arg57 and Lys161. In this process, the van der Waals and electrostatic contributions from the interactions between ATP and the UMP kinase prevail over the unfavorable desolvation energy of ATP, resulting in a favorable binding process and yielding the lowest energy state corresponding to the product structure.

### The allosteric mechanism for ATP molecules

**Figure 7:**
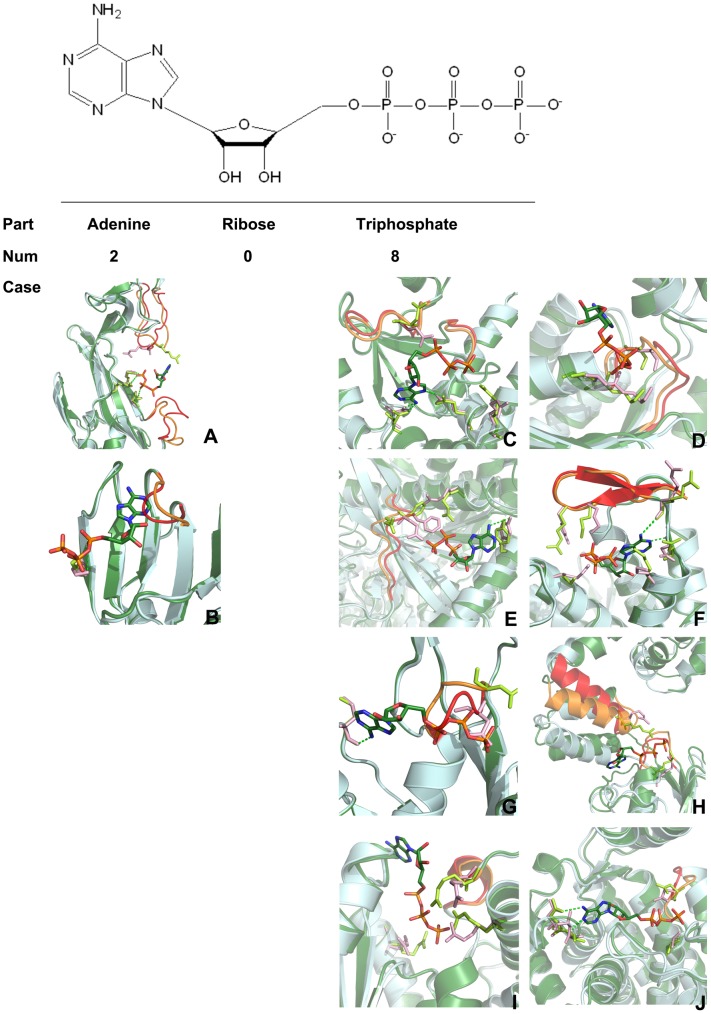
Structural identification of allosteric triggers of an allosteric molecule. The crystal structures of allosteric ATP unbound and bound proteins are shown in pale cyan and dark green, respectively. The relatively large conformational changes in the allosteric ATP unbound and bound proteins are shown in red and orange, respectively, coupled with the residues in light pink and light green, respectively. The adenine in ATP is an allosteric trigger in two cases: (A) P2X_4_ ion channel (PDB: 4DW0 vs. 4DW1; the former is allosteric ATP unbound structure and the latter is allosteric ATP bound structure); and (B) Aspartate carbamoyltransferase (PDB: 6AT1 vs. 4AT1). The triphosphate part of ATP is an allosteric trigger in eight cases: (C) Cytosolic 5’-nucleotidase II (PDB: 2XCX vs. 2XCW); (D) ClpX (PDB: 3HTE vs. 3HWS); (E) Glycogen phosphorylase (PDB: 1FC0 vs. 1FA9); (F) Ribonucleotide reductase (PDB: 1R1R vs. 3R1R); (G) MutS (PDB: 1E3M vs 1W7A); (H) DnaA (PDB: 1L8Q vs. 2HCB); (I) Phosphofructokinase 1 (PDB: 2PFK vs. 1PFK); (J) Chaperonin GroEL (PDB: 1KP0 vs. 1KP8).

Although the above analyses elucidated the binding sites and the related binding process of allosteric ATP molecules, the mechanism of allostery for these identical ATP molecules in different allosteric sites of proteins remains unclear. According to the structural view of allostery [30], [31], allostery works by the propagation of strain energy created at the allosteric site by ligand binding, post-translation modifications, or mutations of the functional site. Guided by this principle, we investigated which part of ATP (the adenine, ribose, or triphosphate) triggers allostery. Therefore, we first analyzed the detailed interactions between each part of ATP and the corresponding protein. As shown in Table 2, the analysis revealed that the contributions of the interactions stem mainly from the adenine and triphosphate parts of ATP. Subsequently, a comprehensive structural analysis of the identical protein between the allosteric ATP-bound and unbound structures was performed [32]. Ten out of thirteen allosteric ATP unbound structures were retrieved from the PDB. A protein 3D structural alignment of the allosteric ATP-bound and unbound structures (Figure 7) displayed that the local structure of proteins proximal to the adenine portion of ATP shows significant conformational change with limited conformational change proximal to the ribose and triphosphate parts of ATP in two cases (Figure 7A–B). This finding is indicative of an allosteric trigger by the adenine part of ATP. By contrast, the local structure of the proteins shows significant conformational change in the proximity of the triphosphate portion of ATP with limited conformational change in the proximity of the adenine and ribose portions of ATP in the remaining eight cases (Figure 7C–J). This finding is indicative of a trigger by the triphosphate part of ATP.

**Table 2:** Interactions between adenine, ribose, and triphosphate parts of ATP and allosteric proteins.

PDB entry	Adenine		Ribose	Triphosphate
	H-bonds	Hydrophobic	H-bonds	H-bonds/Electrostatic
1FA9	Y75	/	/	R193, D227, R242, R309, R310
1I2D	K527	F446, F529	/	K409, D434, R437, R451, I477
1KP8	N479, A480	P33, I493	G415, D495	G32, D87, G88, T89, T90, T91
1PFK	/	K214^A^,Y55^B^	/	R154^A^, K213^A^, R21^B^, R25^B^, R54^B^
1W7A	I597	F596, H760	/	M617, G619, K620, S621, T622, E694
2HCB	F88	I89	H127	G124, K125, T126, H127, D181, R277
2JJX	N107^A^, A110^A^	E111^A^, I114^B^		R99^A^, R117^A^, Y126^A^, R117^B^, H120^B^
2XCW	N154, Q453	F354, I152	/	R144, D145, R456, Y457
3HWS	I79	L127, L317, I325	/	G122, S123, G124, K125, T126, R370
3R1R	E15, R16, N18	V7, I17, I22	I22	K9, E15, K21, T55, K91
4AT1	I12, K60, Y89	V91	/	K94
4DW1	/	R143	/	N296, R298, K316
4GFH	N99	I104, R77	S127, S128	S127, N129, R141, K147, K367, Q365

For example, in the P2X_4_ ion channel (Figure S4A), residues Asn296, Arg298, and Lys316 from both the unbound (PDB: 4DW0) and ATP-bound (PDB: 4DW1) structures engage in hydrogen bonding/electrostatic interactions with the triphosphate in ATP, revealing marginal conformational changes proximal to the triphosphate in ATP. However, the conformation of Arg143 in the two structures shows prominent variation, giving rise to the markedly local conformational change between the head and dorsal-fin domains proximal to the adenine portion of ATP. This local conformational change is transmitted from the lower body domain to transmembrane domains 1 (TM1) and TM2, and leads to the opening of the ion channel pore because of the outward flexing of TM1 and TM2 [19]. In addition, biochemical experiments demonstrated that GTP and UTP are unable to activate P2X_4_ receptors [33], further supporting the notion that the adenine portion of ATP is the trigger of allostery. In the case of human liver glycogen phosphorylase (Figure S4B), Tyr75 from both the unbound (PDB: 1FC0, inactive state) and ATP-bound (PDB: 1FA9, active state) docked structures forms hydrogen bonding interactions with the adenine in ATP, revealing marginal conformational changes in the proximity of the adenine in ATP. However, residues Glu195, Phe196, and Arg309 proximal to the triphosphate in ATP show pronounced conformational changes, which propagate to the active site to switch the gate (residues 280–289) from the “closed” position to the “open” position, allowing glycogen and pyridoxal phosphate access to the catalytic site [34].

## Discussion

ATP is generally utilized by protein kinases or enzymes as the source of phosphate groups to phosphorylate their substrates. Thus, ATP commonly acts as a substrate by virtue of its hydrolysis. Conversely, compared with the overwhelming number of protein-substrate ATP complexes, the few proteins capitalize on ATP as an allosteric modulator to regulate their functional activity. This may be ascribed to the dearth of ATP allosteric sites in protein surfaces to accommodate ATP binding. Simultaneously, the question arises as to why the co-evolution of ATP and optimized allosteric sites did not occur on a more extensive scale. Evolution seemingly selected for other allosteric regulatory mechanisms such as allosteric post-translational modifications [35]. The commonality of the allosteric phosphorylation mechanism may indicate the advantages of a covalent linkage which also allows a higher residence time.

According to ASD v2.0 [15], allosteric proteins in which ATP has been biologically confirmed to serve as an allosteric modulator are 78 (Table S6) regardless of species. Taking into account the species, the available co-crystal structures of proteins-allosteric ATP complexes, and the structural diversity of co-crystal structures, only thirteen distinct co-crystal structures of protein-allosteric ATP or its derivative complexes are available. In humans, all of these allosteric proteins are located inside the cells with the exception of the P2X_4_ receptor which is expressed on the cell surface. These data suggest that ATP can also act as an allosteric modulator to regulate protein function inside cells. As to extracellular regulation, all P2X subtypes (P2X_1-7_) are membrane ion channels that allosterically respond to the binding of extracellular ATP (only the crystal structure of the P2X_4_ receptor-allosteric ATP complex has been described) [19]. The physiological functions of the seven P2X receptor subtypes include modulation of synaptic transmission, contraction of smooth muscle, secretion of chemical transmitters and the regulation of the immune response [18]. ATP drugs, mostly as adenosine disodium triphosphate tablets, are frequently used in the treatment of progressive muscular atrophy, myocardial diseases, hepatitis (as an adjuvant therapy) and heart failure. Based on the present study, we suggest that extracellular ATP molecules, such as ATP drugs, serve as allosteric modulators in extracellular signaling by activating the P2X receptors, a suggestion supported by the functional roles of ATP drugs corresponding to the pharmacology of P2X receptors [33].

We systematically analyzed the amino acid composition of allosteric and substrate ATP-binding sites. In the substrate ATP-binding sites, the amino acid composition is conserved as a consequence of high sequence-similarities of enzymes. However, the composition of allosteric ATP-binding sites varies. The sequence variability in the allosteric ATP-binding sites is likely to be linked to the fine-tuning of allosteric regulation commensurate with function [36]–[40].

The conformational diversity of allosteric and substrate ATP molecules may reflect the properties of binding sites. Overall, substrate ATP molecules show smaller conformational changes, indicative of the conserved substrate ATP-binding sites of enzymes. In contrast, allosteric ATP molecules show larger conformational changes, indicative of the structural variations of allosteric ATP-binding sites. These differences in structural propensities of ATP may be attributed to the different functional roles of ATP in the allosteric and substrate sites. The structural analysis reveals that all ATP molecules in the substrate binding sites conserve extended conformations whereas ATP molecules in the allosteric binding sites may adopt compact conformations (Figure 3). Among the thirteen allosteric cases, more than half of ATP molecules regulate protein functions with compact conformations encapsulated in deep hydrophobic pockets (ribonucleotide reductase, P2X_4_ ion channel, cytosolic 5’-nucleotidase II, glycogen phosphorylase, phosphofructokinase 1, ATP sulfurylase and ClpX). However, the remaining six ATP molecules in GroEL, MutS, DnaA, DNA Topoisomerase II, UMP kinase and Aspartate carbamoyltransferase function as allosteric modulators with extended conformations, which is similar to ATP conformations in the substrate binding sites. Remarkably, the allosteric binding sites of GroEL, MutS, DnaA and DNA Topoisomerase II have dual effects on allosteric regulation and ATPase activities, ATP molecules in the sites allosterically induce protein functions and then are hydrolyzed to ADP when the allosteric regulations are complete [16], [17], [41], [42]. To carry out both allosteric and substrate functions, ATP molecules thus adopt extended conformations in the sites. In UMP kinase, ATP molecules allosterically bind at the protein interfaces between neighboring subunits. The subunits interfaces are narrow, leading to the bound allosteric ATP molecules in extended conformations. Despite the overall extended conformations, the triphosphate moiety of ATP is in the curved conformation in the allosteric binding site, resulting in the formation of internal hydrogen bonds between the γ-phosphate group of triphosphate and the hydroxyl group of ribose. In aspartate carbamoyltransferase, the allosteric ATP binding site is rather flat in the surface of enzyme, with the adenine and ribose moieties of ATP occupying the cavity and the triphosphate moiety of ATP drifting towards the solvent (Figure S5). The feature of allosteric site and the interaction mode between the enzyme and ATP render the allosteric ATP molecule in extended conformation. Overall, the features of allosteric ATP-binding sites in allosteric proteins and the functional roles of allosteric ATP molecules in allosteric proteins determine the conformations of ATP, compact or extended conformations, in allosteric sites. An MD simulation of ATP in solution indicates that the conformational ensemble of ATP in its unbound state is primarily characterized by two states: the compact conformation and the extended conformation. These two states agree with the structural analysis.

The UMP kinase was selected to explore the dynamic process of the access of ATP to the corresponding allosteric and substrate binding sites because this enzyme possesses both the allosteric and substrate ATP-binding sites [22]. NEB simulations revealed markedly different pathways. Notably, the conformational rollover of ATP was observed in the process of ATP binding to the allosteric site but not to the substrate site. This may reflect the differences in the volume and shape of the allosteric and substrate binding sites of the UMP kinase. As shown in Figure 4B, the UMP kinase substrate ATP-binding sites point toward the solvent, and the cavity is spacious which may facilitate the access of ATP to the UMP kinase substrate binding sites. Conversely, the allosteric ATP-binding sites are located at the subunit interface. As shown in Figure 4B, the γ-phosphate moiety of ATP points toward the solvent, whereas the nucleoside moiety points toward the cavity interior. As a consequence, the cavity of the allosteric binding sites is narrow, hindering the access of ATP to the allosteric binding sites. The calculated barrier of ATP binding to its substrate site is lower than that to the allosteric site (22.8 kcal/mol versus 30.2 kcal/mol, respectively), and the total interaction energy between the UMP kinase and the substrate ATP molecule is also lower than that between the UMP kinase and the allosteric ATP molecule (−59.3 kcal/mol versus −50.8 kcal/mol, respectively). Collectively, these data suggest that, in solution, ATP preferentially binds to substrate sites of the UMP kinase.

Once an allosteric ATP molecule occupies the allosteric binding site of a specific protein, the source of the allosteric trigger from ATP to fuel allosteric communication between allosteric and active sites may differ. Our structural analysis revealed that a majority of proteins (80%) select the triphosphate in ATP as an allosteric trigger, whereas a smaller number of proteins (20%) opt for the adenine in ATP as an allosteric trigger. The significant difference between the contributions of the allosteric trigger from ATP indicates that the conformational flexibility of the triphosphate in ATP endows ATP with various regulatory mechanisms and may play a crucial role in the initiation of allostery.

## Materials and Methods

### ATP allosteric and substrate datasets

We built two types of ATP datasets: allosteric and substrate. First, we constructed an annotated ATP-allosteric dataset collected from a hand-curated dataset. ASD v2.0 has manually curated allosteric proteins and allosteric modulators with at least three cases with experimental evidence, crystal structure of the complex or biochemical data [15]. The non-redundant 3D-structures of allosteric proteins in complex with allosteric ATP and its derivatives were considered. We retrieved 13 allosteric proteins deposited in ASD (Table 1). In the 3D-structures of glycogen phosphorylase (PDB: 1FA9) [34], ATP sulfurylase (PDB: 1I2D) [43], phosphofructokinase 1 (PDB: 1PFK) [44], and ClpX (PDB: 3HWS) [45], the solved allosteric effectors in their ATP binding sites are the ATP derivatives, which are most likely because of the hydrolysis of ATP during crystallization. Therefore, we manually docked the ATP molecule into the allosteric ATP binding site in the aforementioned proteins. Second, we constructed an ATP substrate dataset. Twenty-four co-crystal structures of proteins in complex with substrate ATP molecules (Table S3), which exhibit structural diversity, were extracted from the PDB database [21]. The binding site residues for the allosteric and substrate ATP molecules were identified from those within 6 Å of ATP using a fpocket-based pocket detection algorithm [46]. Surface residues were identified as those with high solvent accessible surface areas (SASAs) (>50% of SASA values for corresponding residues in the natural state), which were calculated by POPS [47].

### Phylogenetic tree analysis

To recapitulate the family profile of allosteric proteins, a phylogenetic tree was built from the ATP allosteric database. A multiple sequence alignment (MSA) was performed by a ‘progressive algorithm’ using ClustalX [48] and BLOSUM 30 matrix [49]. MEGA 5.0 [50] was used to build the phylogenetic tree from the MSA results.

### Sequence evolution analysis

The sequence alignments and the calculation of amino acid conservation scores for substrate, allosteric, and surface residues were carried out via the ConSurf server [51] using default parameters. ConSurf retrieved homologous sequences to calculate amino acid conservation scores from the UniProtKB/SwissProt database [52]. The high sequence identity (>95%) and short sequence length (<60%) to the query sequence were eradicated, with the resulting homologous sequences to calculate amino acid conservation scores. The percentile normalization method to normalize the conservation scores, as done by Yang *et al.* [21], was performed to compare the conserved degree of substrate, allosteric, and surface sites.

### Docking studies

Molecular docking of ATP to the active site of 1FA9, 1I2D, 1PFK, and 3HWS was performed using the AutoDock 4.2 program [53]. Polar hydrogen atoms were added to the proteins. Kollman united partial atomic charges were then assigned and the AutoDock atom types were defined for the proteins using AutoDock Tools (ADT). ATP geometry was minimized using the AM1 Hamiltonian as implemented in the program Gaussian09 [54]. Gasteiger charges were then added to ATP, with the default root, rotatable bonds, and torsion setting for ATP using the TORSDOF module in ADT. The grid center was defined at the centroid of the ATP derivatives in the aforementioned proteins, and the number of grid points in the *x*, *y*, and *z* directions were set to 60, 60, and 60 with a spacing value of 0.375 Å using AutoGrid. The distance-dependent function of the dielectric constant was used to calculate the energetic maps. The Lamarckian genetic algorithm was employed for the ATP conformational search with identical docking parameters used previously [55], [56]. Fifty independent docking runs were conducted, and the binding energy was used to rank the docked ATP in order of fitness.

### Simulation systems

In the simulation of ATP in aqueous solution, the structure of ATP in complex with Mg^2+^, [ATP:Mg]^2−^, was extracted from the UMP kinase of *Bacillus anthracis* (PDB: 2JJX) [22] as previously suggested by Li *et al.* [57] in the simulation of ATP in water solvent. The polyphosphate parameters developed by Carlson *et al.* were adopted for ATP [58]. ATP was explicitly solvated by TIP3P [59] water molecules in a truncated octahedral box. The distance to the edge of the solvent box from the ATP atoms was set to be 15 Å. Counterions were added to maintain electroneutrality in the system. The final system contains ∼7.5×10^3^ atoms.

To perform unbiased simulations of the allosteric and substrate ATP-bound UMP kinase, the bound UMP-ATP complex was modeled on the basis of the crystal structure of the *Bacillus anthracis* UMP kinase (PDB: 2JJX) [22]. In the crystal structure of the *Bacillus anthracis* UMP kinase, the ATP and UMP in the active sites are not clearly visible in the electron density map. Therefore, ATP and UMP were manually docked into the active sites of the *Bacillus anthracis* UMP kinase after superposition with the *Pyrococcus furiosus* UMP kinase structure that was solved in complex with UMP and AMP-PCP (PDB: 2BMU) [28]. The unbound state of the *Bacillus anthracis* UMP kinase was obtained by removing both allosteric and substrate ATP molecules from the crystal structure. Prior to hydrogen atom placement, the program PROPKA [60] was used to perform p*K*_a_ calculations to aid the assignment of side chain protonation states of all His residues. The AMBER ff03 force field [61] was assigned for proteins. ATP parameters used with the AMBER force field were included [58], and UMP parameters were calculated with the RESP HF/6-31G* method using the Antechamber encoded in AMBER11 [62] and Gaussian09 [54]. Both systems were then solvated in ∼158×159×106 Å water boxes with TIP3P water molecules to ensure the minimum distance between any protein atom and the side of the box is 25 Å and neutralized with 25 (3) Na^+^ ions for the bound (unbound) complex. The final systems contained ∼2.4×10^5^ atoms.

### MD simulations

To remove bad contacts in the solvated systems for the MD simulations, the steepest descent and conjugate gradient algorithm energy minimization methods were used. Energy minimization of the water molecules and counterions with a positional restraint of 500 kcal mol^−1^ Å^−2^ in the complex was first performed; the steepest descent method was applied for the first 2,000 steps, and then the conjugated gradient method was used for the subsequent 3,000 steps. Afterward, the entire system was minimized without any restraints; the steepest descent method was used for the first 4,000 steps, and then the conjugated gradient method was used for the subsequent 6,000 steps. After minimization, each system was heated gradually from 0 K to 300 K within 200 ps. This was followed by constant temperature equilibration at 300 K for 500 ps, with a positional restraint of 10 kcal mol^−1^ Å^−2^ in the complex in a canonical ensemble (NVT). The structures from the final stage of equilibration were chosen as the initial conformations for subsequent MD simulations.

A total of 2 microsecond (µs) MD simulations were performed for the simulation of ATP in a fully solvated water environment, and each of 100 ns MD simulations was conducted for the ATP bound and unbound UMP kinase (Table S7). All simulations were performed with periodic boundary conditions using the NPT ensemble. Langevin dynamics [63] was used to maintain the temperature at 300 K with a collision frequency of 1 ps^−1^, and a Langevin piston was assigned to maintain the pressure at 1 atm. An integration step of 2 fs was set for the MD simulations. The long-range electrostatic interactions were incorporated by using the particle mesh Ewald method [64] with a cubic fourth-order B-spline interpolation and by setting the direct sum tolerance to 10^−5^. A cut-off equal to 10 Å was used for short-range electrostatics and van der Waals interactions. The SHAKE method [65], with a tolerance of 10^−5^ Å, was applied to constrain all covalent bonds that involve hydrogen atoms.

After MD simulations, the analysis of RMSD of UMP kinase relative to the initial structure, together with the calculations of the temperature, total energy, mass density, and volume during MD simulations (Figure S3), suggests that the 100 ns MD simulations are sufficient for obtaining the stability of UMP-ATP complexes.

### Cluster analysis

To generate representative structural ensembles for the ATP unbound UMP state, a RMSD conformational clustering was performed. The cluster analysis was undertaken to group coordinate snapshots from the trajectory into distinct sets by virtue of the PTRAJ module in AMBER 11. The clustering was performed with the average-linkage algorithm that has been described previously [66]. Structures were selected in 200 ps intervals over the simulation trajectory of the unbound state. The resulting 500 structures were superimposed using all C_a_ atoms to remove overall rotation and transition. Then, pairwise C_α_ atoms RMSD comparisons were performed between any snapshot and the average coordinate after rigid-body alignment using a threshold of 1.5 Å. Two main clusters were obtained. One snapshot was chosen from each cluster for NEB calculations.

### Nudged elastic band (NEB) method

The NEB method [67]–[70] is a powerful algorithm to investigate the transition pathway for the binding of ATP to its allosteric and substrate binding sites. In NEB, a string of replicas (or ‘images’) of the system are created and connected together with springs to form a discrete representation of a path from the start to end configuration. Minimization of the entire system with the start and the end point structures fixed provides a minimum energy path. Each image between the two point structures is connected to the previous and next image by ‘springs’ along the path that maintain each image from sliding down the energy landscape onto adjacent images. In NEB, the total force *F* on each image, *i*, is decoupled as a perpendicular and parallel force by a tangent vector [Eq.(1)]. The perpendicular component of the force is obtained by subtracting out the parallel component of the force [Eq. (2)], where ∇*V*(*P_i_*) is the gradient of the energy with respect to the atomic coordinates in the system at image, *i*, and τ is the 3N dimensional tangent unit vector that describes the path. The parallel component of the force accounts for the artificial springs linking each image together [Eq. (3)], where *k_i_* is equal to the spring constant between images *P_i_* and *P_i-1_*, and *P* is the 3N dimensional position vector of image *i*. 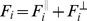
(1)

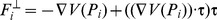
(2)

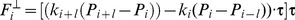
(3)


In all NEB calculations, the end point was selected from the equilibrated ATP bound UMP kinase. The starting point was the source ATP unbound UMP kinase. We placed ATP in the bulk in which the minimum distance between ATP and UMP kinase was larger than 15 Å with different initial positions. Ten different sets of ATP configurations were chosen from the ATP clusters (half from the compact and half from the extended clustering). To further enhance the sampling, two distinct unbound UMP kinase structures were used based on the conformational diversity of the unbound UMP kinase state in the MD simulations for each ATP configuration. Therefore, all twenty NEB calculations were performed to explore the dynamics process of ATP binding to the allosteric and substrate sites of the UMP kinase.

The simulated annealing version of NEB from AMBER 11 was applied in these simulations. The initial NEB pathway consisted of eleven staring-points followed by eleven end-points. The initial path was heated from 0 K to 300 K in 100 ps with a Langevin dynamics of frequency of 1000 ps^−1^ and a spring force of 10 kcal mol^−1^ Å^−2^. Then, the path was equilibrated at 300 K in 200 ps. After that, a total of 600 ps simulated annealing protocol (Table S8) involved quickly heating the path to 500 K, followed by slow cooling and finally quenched the dynamics to remove any remaining kinetic energy from the path with the spring force of 50 kcal/mol [71], [72]. The random number generator was seeded differently for each calculation.

## Supporting Information

Figure S1 Distributions of RMSD values for ATP in the substrate and allosteric datasets. The statistical significant (*P*-value) was calculated by the Mann-Whitney U test.
(TIF)

Figure S2 Comparison of conservation scores for residues in the allosteric and substrate ATP-binding sites and surface in the UMP kinase family. The statistical significant (*P*-value) was calculated by the Mann-Whitney U test.
(TIF)

Figure S3 The time-dependences of Cα atoms RMSD of UMP kinase, temperature, total energy, mass density, and volume during 100 ns MD simulations. The interaction energies between UMP kinase and ATP were calculated every 10 ns interval trajectories. The error bars represent standard deviations.
(TIF)

Figure S4 The allosteirc trigger of the adenine in ATP in the P2X_4_ ion channel (A) and the triphosphate in ATP in the glycogen phosphorylase (B). The structural features and color scheme follow the description in Figure 7. The functional sites for TM1 and TM2 of the P2X_4_ ion channel and the gate (residues 280–289) of the glycogen phosphorylase are highlighted.
(TIF)

Figure S5 The locations of the allosteric ATP-binding sites in the aspartate carbamoyltransferase.
(TIF)

Table S1 Sequence identity (%) between the 13 allosteric proteins.
(DOC)

Table S2 Root-mean-square deviation (Å) between the 13 allosteric proteins.
(DOC)

Table S3 Twenty-four proteins modulated by substrate ATP.
(DOC)

Table S4 The conservation scores for residues in the allosteric (10 residues) and substrate (22 residues) ATP-binding sites and surface (31 residues) in the UMP kinase family.
(DOC)

Table S5 The energy barriers (kcal/mol) of ATP access to allosteric and substrate binding sites for each NEB simulation.
(DOC)

Table S6 Proteins allosterically modulated by ATP deposited in ASD v2.0.
(DOC)

Table S7 Summary of MD simulation systems.
(DOC)

Table S8 Simulated annealing protocol used for minimization in NEB simulations.
(DOC)
