# Supplementary figures and images for: The Structural Basis of ATP as an Allosteric Modulator
Source: PLoS Comput Biol. 2014 Sep 11;10(9):e1003831. doi: 10.1371/journal.pcbi.1003831 (PMC4161293; doi:10.1371/journal.pcbi.1003831)

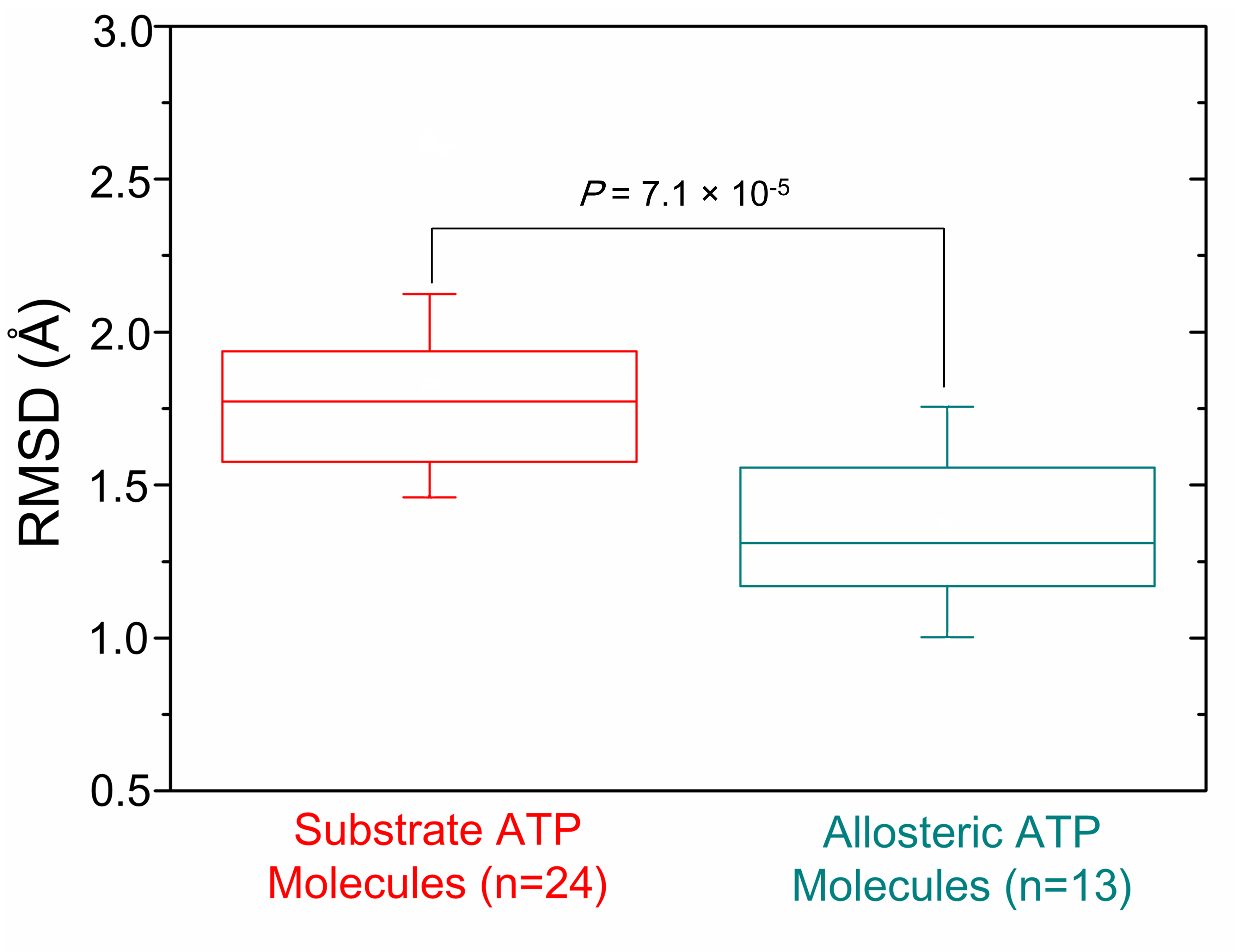

Supplement: Figure S1 — Distributions of RMSD values for ATP in the substrate and allosteric datasets. The statistical significant (P-value) was calculated by the Mann-Whitney U test. (TIF) [file pcbi.1003831.s001.tif]

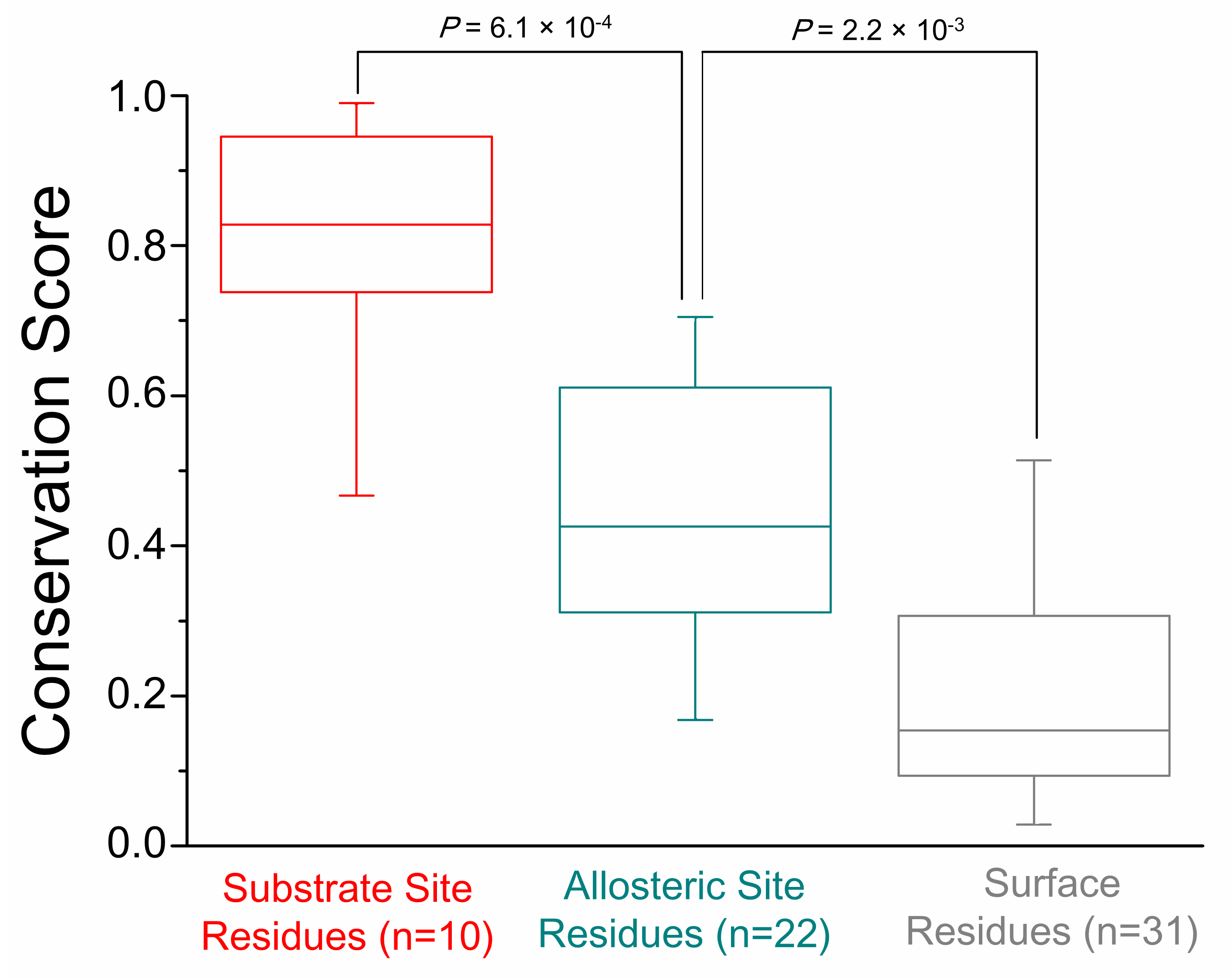

Supplement: Figure S2 — Comparison of conservation scores for residues in the allosteric and substrate ATP-binding sites and surface in the UMP kinase family. The statistical significant (P-value) was calculated by the Mann-Whitney U test. (TIF) [file pcbi.1003831.s002.tif]

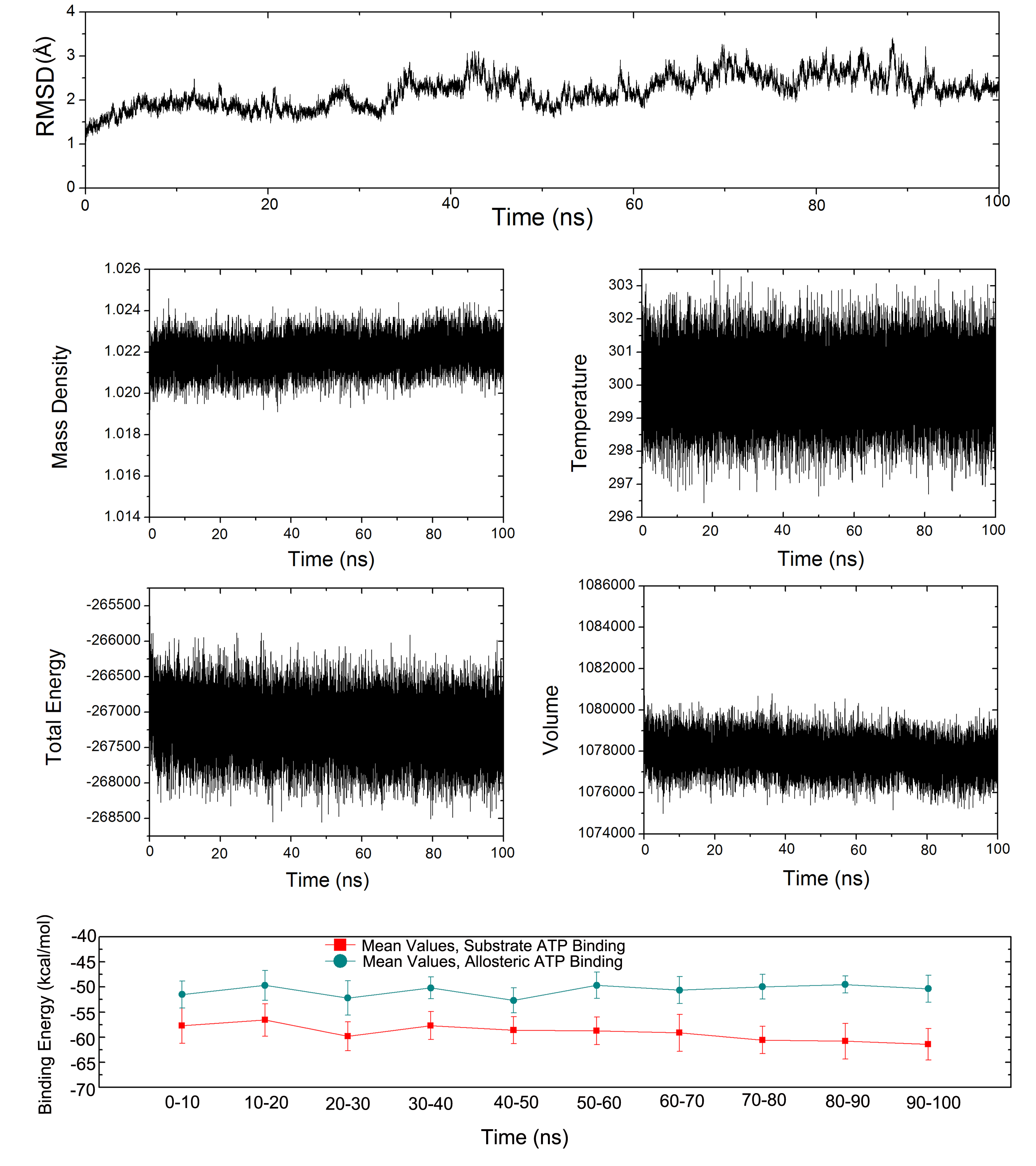

Supplement: Figure S3 — The time-dependences of Cα atoms RMSD of UMP kinase, temperature, total energy, mass density, and volume during 100 ns MD simulations. The interaction energies between UMP kinase and ATP were calculated every 10 ns interval trajectories. The error bars represent standard deviations. (TIF) [file pcbi.1003831.s003.tif]

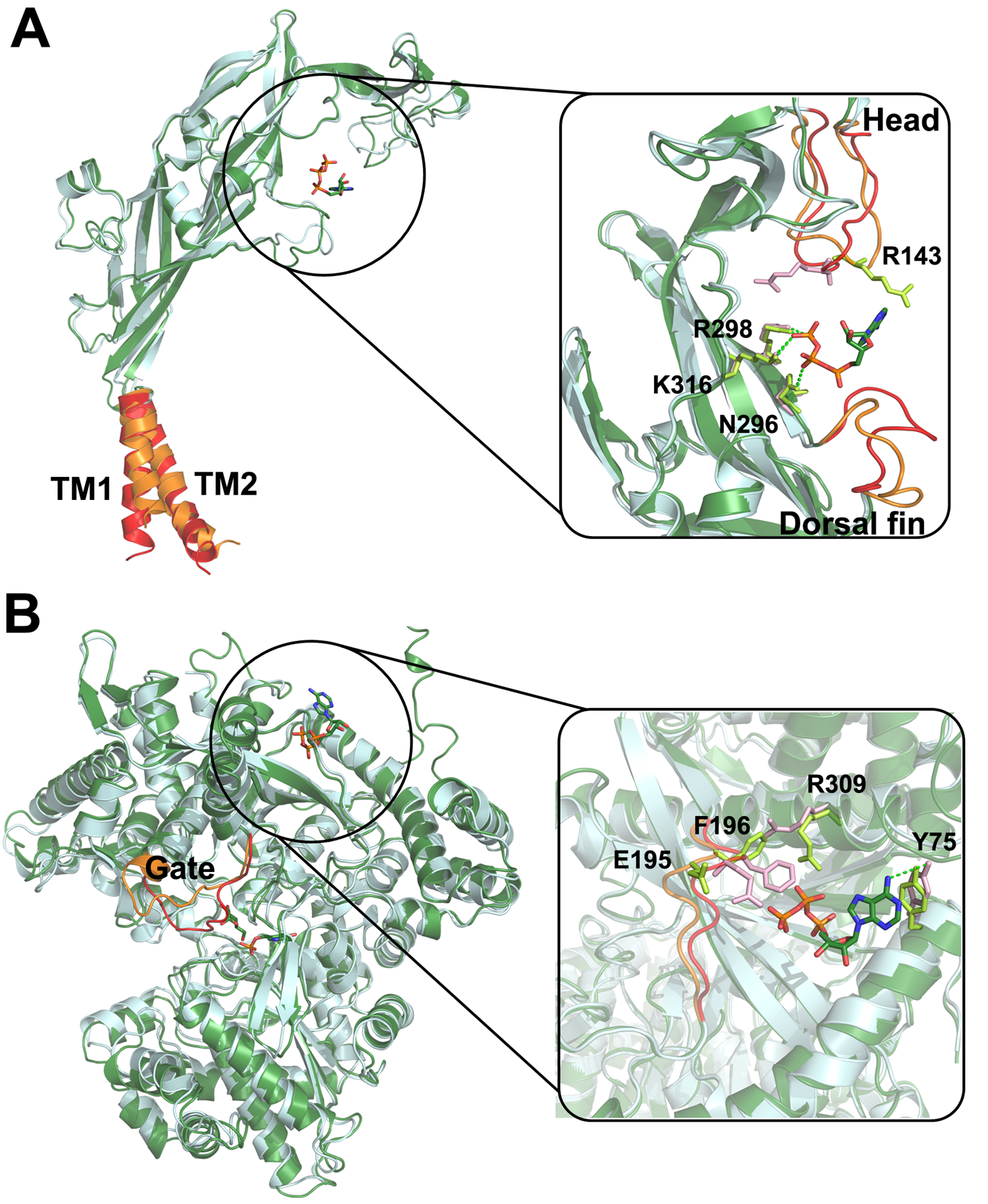

Supplement: Figure S4 — The allosteirc trigger of the adenine in ATP in the P2X4 ion channel (A) and the triphosphate in ATP in the glycogen phosphorylase (B). The structural features and color scheme follow the description in Figure 7. The functional sites for TM1 and TM2 of the P2X4 ion channel and the gate (residues 280–289) of the glycogen phosphorylase are highlighted. (TIF) [file pcbi.1003831.s004.tif]

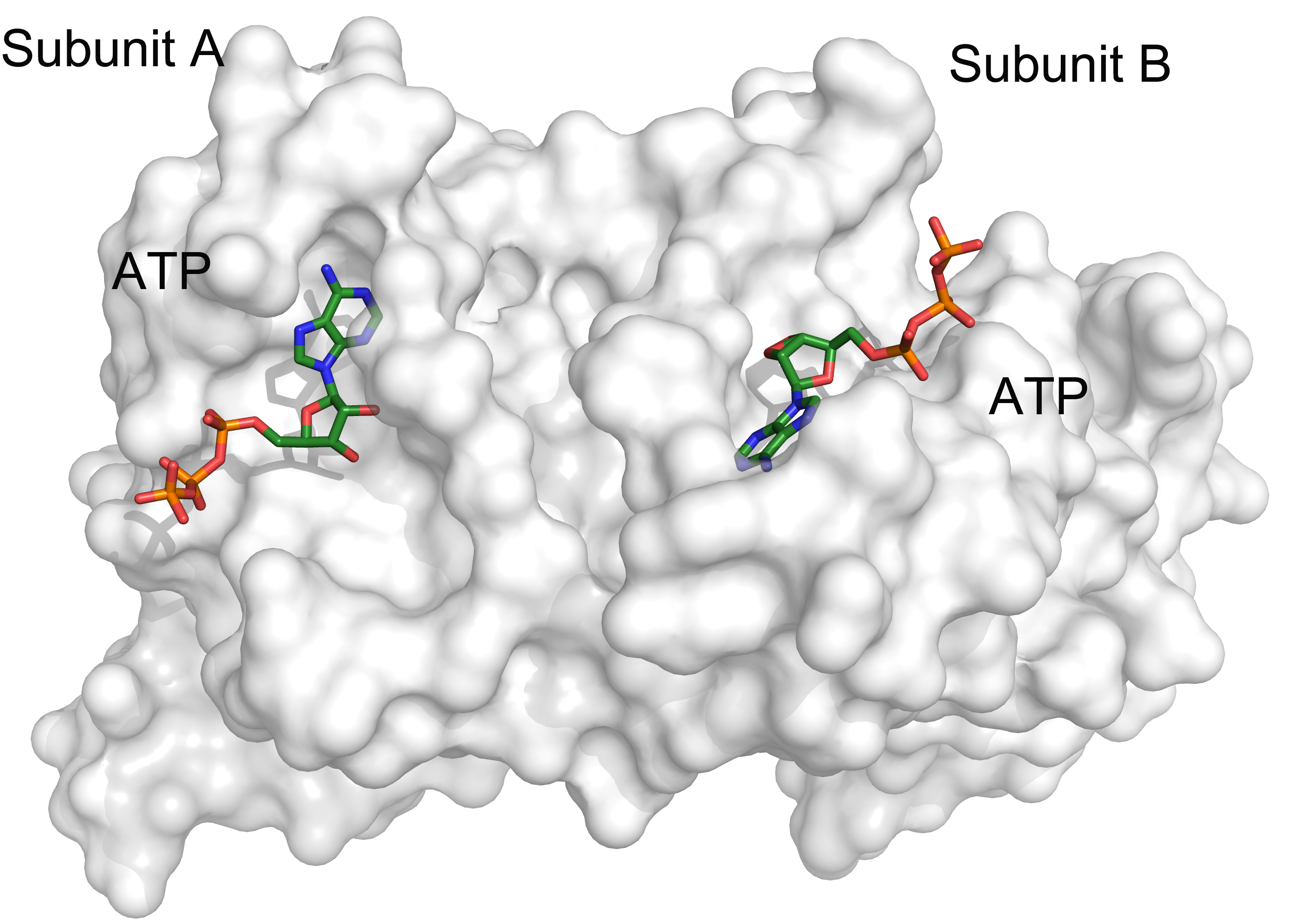

Supplement: Figure S5 — The locations of the allosteric ATP-binding sites in the aspartate carbamoyltransferase. (TIF) [file pcbi.1003831.s005.tif]
