# Supplementary material for: The Structural Basis of ATP as an Allosteric Modulator
Source: PLoS Comput Biol. 2014 Sep 11;10(9):e1003831. doi: 10.1371/journal.pcbi.1003831 (PMC4161293; doi:10.1371/journal.pcbi.1003831)
Supplement: Table S1 — Sequence identity (%) between the 13 allosteric proteins. (DOC) [file pcbi.1003831.s006.doc]

**Table S1:** Sequence identity (%) between the 13 allosteric proteins

| *PDB entry* | *4AT1* | *1I2D* | *2XCW* | *1FA9* | *1KP8* | *3R1R* | *2JJX* | *1PFK* | *3HWS* | *1W7A* | *4GFH* | *2HCB* | *4DW1* |
| --- | --- | --- | --- | --- | --- | --- | --- | --- | --- | --- | --- | --- | --- |
| 4AT1 | 100 | 29.5 | 25.3 | 36.3 | 26.7 | 34.9 | 19.2 | 22.6 | 28.8 | 28.8 | 35.6 | 24.1 | 23.3 |
| 1I2D | 29.5 | 100 | 18.3 | 21.9 | 13.3 | 17.2 | 26.6 | 26.2 | 23.4 | 21.7 | 26.3 | 22.7 | 18.6 |
| 2XCW | 25.3 | 18.3 | 100 | 23.5 | 12.0 | 22.7 | 23.8 | 17.6 | 19.4 | 23.3 | 24.6 | 17.0 | 17.1 |
| 1FA9 | 36.3 | 21.9 | 23.5 | 100 | 23.0 | 13.6 | 29.1 | 30.9 | 27.5 | 15.2 | 17.3 | 27.4 | 22.5 |
| 1KP8 | 26.7 | 13.1 | 12.0 | 23.0 | 100 | 19.7 | 22.5 | 22.2 | 22.5 | 20.4 | 22.9 | 24.3 | 23.8 |
| 3R1R | 34.9 | 17.2 | 22.7 | 13.6 | 19.7 | 100 | 28.3 | 29.5 | 29.2 | 17.0 | 20.4 | 28.4 | 25.6 |
| 2JJX | 19.2 | 26.6 | 23.8 | 29.1 | 22.5 | 28.3 | 100 | 18.5 | 18.9 | 28.7 | 28.3 | 18.9 | 16.9 |
| 1PFK | 22.6 | 26.2 | 17.6 | 30.9 | 22.2 | 29.5 | 18.5 | 100 | 13.3 | 27.9 | 30.9 | 12.4 | 12.1 |
| 3HWS | 28.8 | 23.4 | 19.4 | 27.5 | 22.5 | 29.2 | 18.9 | 13.3 | 100 | 30.3 | 31.6 | 16.0 | 11.3 |
| 1W7A | 28.8 | 21.7 | 23.3 | 15.2 | 20.4 | 17.2 | 28.7 | 27.9 | 30.3 | 100 | 20.8 | 28.7 | 23.8 |
| 4GFH | 35.6 | 26.3 | 24.6 | 17.3 | 22.9 | 20.4 | 28.3 | 30.9 | 31.6 | 20.8 | 100 | 32.5 | 31.0 |
| 2HCB | 24.1 | 22.7 | 17.0 | 27.4 | 24.3 | 28.4 | 18.9 | 12.4 | 16.0 | 28.7 | 32.5 | 100 | 11.5 |
| 4DW1 | 23.3 | 17.6 | 17.1 | 22.5 | 23.8 | 25.6 | 16.9 | 12.1 | 11.3 | 23.8 | 31.0 | 11.5 | 100 |
