# Supplementary material for: The Structural Basis of ATP as an Allosteric Modulator
Source: PLoS Comput Biol. 2014 Sep 11;10(9):e1003831. doi: 10.1371/journal.pcbi.1003831 (PMC4161293; doi:10.1371/journal.pcbi.1003831)
Supplement: Table S2 — Root-mean-square deviation (Å) between the 13 allosteric proteins. (DOC) [file pcbi.1003831.s007.doc]

**Table S2:** Root-mean-square deviation (Å) between the 13 allosteric proteins

| *PDB entry* | *4AT1* | *1I2D* | *2XCW* | *1FA9* | *1KP8* | *3R1R* | *2JJX* | *1PFK* | *3HWS* | *1W7A* | *4GFH* | *2HCB* | *4DW1* |
| --- | --- | --- | --- | --- | --- | --- | --- | --- | --- | --- | --- | --- | --- |
| 4AT1 | 0 | 23.9 | 24.1 | 24.8 | 27.1 | 26.8 | 15.7 | 21.3 | 16.6 | 28.3 | 34.6 | 18.8 | 25.7 |
| 1I2D | 23.9 | 0 | 32.3 | 29.4 | 25.5 | 29.3 | 25.1 | 25.6 | 22.2 | 30.3 | 37.3 | 23.3 | 33.5 |
| 2XCW | 24.1 | 32.3 | 0 | 27.6 | 25.9 | 24.9 | 20.6 | 22.4 | 21.9 | 30.7 | 42.0 | 22.6 | 28.5 |
| 1FA9 | 24.8 | 29.4 | 27.6 | 0 | 29.2 | 26.2 | 27.6 | 29.1 | 25.0 | 35.1 | 41.2 | 22.1 | 33.6 |
| 1KP8 | 27.1 | 25.6 | 25.9 | 29.2 | 0 | 26.8 | 26.6 | 25.1 | 26.1 | 30.8 | 46.7 | 27.7 | 33.0 |
| 3R1R | 26.8 | 29.3 | 24.9 | 26.2 | 26.8 | 0 | 26.4 | 25.4 | 25.6 | 35.3 | 36.4 | 28.8 | 29.9 |
| 2JJX | 15.7 | 25.1 | 20.6 | 27.6 | 26.6 | 26.4 | 0 | 21.7 | 20.9 | 29.5 | 41.2 | 19.5 | 24.3 |
| 1PFK | 21.3 | 25.6 | 22.4 | 29.1 | 25.1 | 25.4 | 21.7 | 0 | 25.5 | 34.1 | 37.8 | 21.1 | 25.5 |
| 3HWS | 16.6 | 22.2 | 21.9 | 25.0 | 26.1 | 25.6 | 20.9 | 25.5 | 0 | 30.8 | 40.4 | 17.5 | 28.9 |
| 1W7A | 28.3 | 30.3 | 30.7 | 35.1 | 30.8 | 35.2 | 29.5 | 34.1 | 30.8 | 0 | 45.6 | 32.5 | 38.3 |
| 4GFH | 34.6 | 37.3 | 42.0 | 41.2 | 46.7 | 36.4 | 41.2 | 37.8 | 40.4 | 45.6 | 0 | 35.3 | 51.0 |
| 2HCB | 18.8 | 23.3 | 22.6 | 22.1 | 27.7 | 28.8 | 19.5 | 21.1 | 17.5 | 32.5 | 35.3 | 0 | 28.2 |
| 4DW1 | 25.7 | 34.0 | 28.5 | 33.6 | 33.0 | 29.9 | 24.3 | 25.5 | 28.9 | 38.3 | 51.0 | 28.2 | 0 |
