# Supplementary material for: The Structural Basis of ATP as an Allosteric Modulator
Source: PLoS Comput Biol. 2014 Sep 11;10(9):e1003831. doi: 10.1371/journal.pcbi.1003831 (PMC4161293; doi:10.1371/journal.pcbi.1003831)
Supplement: Table S3 — Twenty-four proteins modulated by substrate ATP. (DOC) [file pcbi.1003831.s008.doc]

**Table S3:** Twenty-four proteins modulated by substrate ATP

| PDB ID | Resolution  (Å) | Protein name | Organism | EC No. |
| --- | --- | --- | --- | --- |
| 1A82 | 1.80 | Dethiobiotin synthetase BioD 1 | Escherichia coli | 6.3.3.3 |
| 1CM8 | 1.40 | MAP kinase 12 | Homo sapiens | 2.7.11.24 |
| 1E79 | 2.40 | ATP synthase subunit alpha, mitochondrial | Bos taurus | 3.6.3.14 |
| 1MO8 |  | Sodium/Potassium-Transporting ATPase alpha-1 | Rattus norvegicus | 3.6.3.9 |
| 1O93 | 3.49 | S-adenosylmethionine synthase isoform type-1 | Rattus norvegicus | 2.5.1.6 |
| 1OL6 | 3.00 | Aurora kinase A | Homo sapiens | 2.7.11.1 |
| 1QMZ | 2.20 | Cyclin-dependent kinase 2 | Homo sapiens | 2.7.11.22 |
| 1XMI | 2.25 | Cystic fibrosis transmembrane conductance regulator | Homo sapiens | 3.6.3.49 |
| 1XSC |  | Bis(5'-nucleosyl)-tetraphosphatase | Homo sapiens | 3.6.1.17 |
| 2AQX | 2.50 | Inositol 1,4,5-trisphosphate 3-kinase B | Mus musculus | 2.7.1.127 |
| 2B6F |  | Sulfiredoxin | Homo sapiens | 1.8.98.2 |
| 2C8V | 2.50 | Nitrogenase iron protein 1 | Azotobacter vinelandii | 1.18.6.1 |
| 2KMX |  | Copper-transporting ATPase 1 | Homo sapiens | 3.6.3.54 |
| 2OLR | 1.60 | Phosphoenolpyruvate carboxykinase | Escherichia coli | 4.1.1.49 |
| 2W00 | 2.60 | Type I restriction enzyme EcoR124II R protein | Escherichia coli | 3.1.21.3 |
| 2YXU | 2.20 | Pyridoxal kinase | Homo sapiens | 2.7.1.35 |
| 2ZAN | 3.00 | Vacuolar protein sorting-associating protein 4B | Mus musculus | 3.6.4.6 |
| 2ZDQ | 2.30 | D-alanine--D-alanine ligase | Thermus thermophilus | 6.3.2.4 |
| 3A8T | 2.37 | Adenylate isopentenyltransferase | Humulus lupulus | 2.5.1.27 |
| 3AR4 | 2.15 | Sarcoplasmic/endoplasmic reticulum calcium ATPase 1 | Oryctolagus cuniculus | 3.6.3.8 |
| 3C16 | 2.87 | Adenylate cyclase type 5 | Canis lupus familiaris | 4.6.1.1 |
| 3C5E | 1.60 | Acyl-coenzyme A synthetase ACSM2A, mitochondrial precursor | Homo sapiens | 6.2.1.2 |
| 3CRC | 3.00 | Protein mazG | Escherichia coli | 3.6.1.8 |
| 3INN | 2.10 | Pantothenate synthetase | Brucella melitensis | 6.3.2.1 |
