# Supplementary material for: The Structural Basis of ATP as an Allosteric Modulator
Source: PLoS Comput Biol. 2014 Sep 11;10(9):e1003831. doi: 10.1371/journal.pcbi.1003831 (PMC4161293; doi:10.1371/journal.pcbi.1003831)
Supplement: Table S4 — The conservation scores for residues in the allosteric (10 residues) and substrate (22 residues) ATP-binding sites and surface (31 residues) in the UMP kinase family. (DOC) [file pcbi.1003831.s009.doc]

**Table S4:** The conservation scores for residues in the allosteric (10 residues) and substrate (22 residues) ATP-binding sites and surface (31 residues) in the UMP kinase family

| *Allosteric sites* | |  | *Substrate sites* | |  | *Surface sites* | |
| --- | --- | --- | --- | --- | --- | --- | --- |
| Residue  types | Conservation scores |  | Residue types | Conservation scores |  | Residue types | Conservation scores |
| Arg99 | 0.705 |  | Lys10 | 0.928 |  | Arg2 | 0.127 |
| Asn107 | 0.168 |  | Leu11 | 0.467 |  | Pro3 | 0.090 |
| Ala108 | 0.258 |  | Ser12 | 0.939 |  | Lys5 | 0.205 |
| Ala110 | 0.652 |  | Gly13 | 0.978 |  | Thr20 | 0.154 |
| Pro112 | 0.311 |  | Gly51 | 0.973 |  | Gly21 | 0.066 |
| Ile114 | 0.611 |  | Arg57 | 0.990 |  | Asn22 | 0.143 |
| Leu116 | 0.428 |  | Thr140 | 0.891 |  | Ser26 | 0.176 |
| Arg117 | 0.357 |  | Asp142 | 0.945 |  | Arg57 | 0.990 |
| His120 | 0.459 |  | Val159 | 0.809 |  | His59 | 0.279 |
| Tyr126 | 0.434 |  | Ala160 | 0.738 |  | Glu62 | 0.307 |
|  |  |  | Lys161 | 0.914 |  | Trp64 | 0.094 |
|  |  |  | Gln162 | 0.658 |  | Asn73 | 0.514 |
|  |  |  | Val164 | 0.957 |  | Asn95 | 0.254 |
|  |  |  | Gly166 | 0.951 |  | Phe138 | 0.406 |
|  |  |  | Val167 | 0.744 |  | Asn153 | 0.135 |
|  |  |  | Phe168 | 0.795 |  | Asp171 | 0.945 |
|  |  |  | Thr169 | 0.316 |  | Lys173 | 0.098 |
|  |  |  | Ser170 | 0.221 |  | His174 | 0.193 |
|  |  |  | Ile194 | 0.828 |  | Lys176 | 0.078 |
|  |  |  | Gln195 | 0.154 |  | Ser177 | 0.074 |
|  |  |  | Val196 | 0.787 |  | Asn193 | 0.209 |
|  |  |  | Met197 | 0.861 |  | Gln195 | 0.154 |
|  |  |  |  |  |  | Gln199 | 0.373 |
|  |  |  |  |  |  | Tyr208 | 0.639 |
|  |  |  |  |  |  | Asn209 | 0.123 |
|  |  |  |  |  |  | Pro220 | 0.029 |
|  |  |  |  |  |  | Leu228 | 0.029 |
|  |  |  |  |  |  | His231 | 0.029 |
|  |  |  |  |  |  | Asp239 | 0.139 |
|  |  |  |  |  |  | Ala240 | 0.287 |
|  |  |  |  |  |  | His245 | 0.506 |
