# Supplementary material for: The Structural Basis of ATP as an Allosteric Modulator
Source: PLoS Comput Biol. 2014 Sep 11;10(9):e1003831. doi: 10.1371/journal.pcbi.1003831 (PMC4161293; doi:10.1371/journal.pcbi.1003831)
Supplement: Table S5 — The energy barriers (kcal/mol) of ATP access to allosteric and substrate binding sites for each NEB simulation. (DOC) [file pcbi.1003831.s010.doc]

**Table S5:** The energy barriers (kcal/mol) of ATP access to allosteric and substrate binding sites for each NEB simulation

| *ATP access to allosteric binding site* | | | |  | *ATP access to substrate binding site* | | | |
| --- | --- | --- | --- | --- | --- | --- | --- | --- |
| Num | Minimum  (kcal/mol) | Maximum  (kcal/mol) | Energy barrier  (kcal/mol) |  | Num | Minimum  (kcal/mol) | Maximum  (kcal/mol) | Energy barrier  (kcal/mol) |
| NEB1 | -3.49 | 24.54 | 28.03 |  | NEB1 | -3.72 | 16.76 | 20.48 |
| NEB2 | -4.82 | 25.64 | 30.46 |  | NEB2 | -3.88 | 18.24 | 22.12 |
| NEB3 | -5.43 | 22.94 | 28.37 |  | NEB3 | -4.36 | 15.91 | 20.27 |
| NEB4 | -3.67 | 26.05 | 29.72 |  | NEB4 | -4.63 | 19.16 | 23.79 |
| NEB5 | -3.32 | 28.80 | 32.12 |  | NEB5 | -3.84 | 16.27 | 20.11 |
| NEB6 | -4.61 | 30.11 | 34.72 |  | NEB6 | -5.07 | 19.48 | 24.55 |
| NEB7 | -2.21 | 25.91 | 28.12 |  | NEB7 | -4.45 | 16.59 | 21.04 |
| NEB8 | -6.33 | 26.36 | 32.69 |  | NEB8 | -5.75 | 19.21 | 24.96 |
| NEB9 | -3.59 | 26.39 | 29.98 |  | NEB9 | -5.42 | 20.54 | 25.96 |
| NEB10 | -3.24 | 25.29 | 28.53 |  | NEB10 | -3.84 | 15.27 | 19.11 |
| NEB11 | -3.94 | 26.76 | 30.70 |  | NEB11 | -4.78 | 18.07 | 22.85 |
| NEB12 | -3.63 | 24.04 | 27.67 |  | NEB12 | -4.45 | 17.88 | 22.33 |
| NEB13 | -4.39 | 25.38 | 29.77 |  | NEB13 | -4.22 | 17.09 | 21.31 |
| NEB14 | -3.91 | 25.46 | 29.37 |  | NEB14 | -6.11 | 18.07 | 24.18 |
| NEB15 | -3.45 | 26.25 | 29.70 |  | NEB15 | -5.23 | 19.02 | 24.25 |
| NEB16 | -4.62 | 26.82 | 31.44 |  | NEB16 | -5.49 | 18.79 | 24.28 |
| NEB17 | -3.90 | 26.30 | 30.20 |  | NEB17 | -4.26 | 17.70 | 21.96 |
| NEB18 | -2.17 | 26.63 | 28.80 |  | NEB18 | -5.02 | 18.25 | 23.27 |
| NEB19 | -3.49 | 24.54 | 28.03 |  | NEB19 | -4.24 | -15.53 | 23.77 |
| NEB20 | -3.83 | 26.53 | 30.36 |  | NEB20 | -5.08 | -16.73 | 21.81 |
