# Supplementary material for: The Structural Basis of ATP as an Allosteric Modulator
Source: PLoS Comput Biol. 2014 Sep 11;10(9):e1003831. doi: 10.1371/journal.pcbi.1003831 (PMC4161293; doi:10.1371/journal.pcbi.1003831)
Supplement: Table S6 — Proteins allosterically modulated by ATP deposited in ASD v2.0. (DOC) [file pcbi.1003831.s011.doc]

**Table S6:** Proteins allosterically modulated by ATP deposited in ASD v2.0

| no. | protein name | organism | | swissprot ID | modulator feature | reference |
| --- | --- | --- | --- | --- | --- | --- |
| 1 | 3-phosphoinositide-dependent protein kinase 1 | | Human | O15530 | Activator | Engel M, et al. EMBO J. 2006,25:5469-5480.;Hindie V, et al. Nat.Chem.Biol. 2009,5:758-764. |
| 2 | 5'-nucleotidase | | Human | P21589 | Inhibitor | Ipata P L, et al. Biochemistry. 1968,7:507-515 |
| 3 | AMP deaminase 1 | | Chicken | P81073 | Regulator | Yoshino M, et al. J Biochem. 80:299-308 |
| 4 | AMP nucleosidase | | Azotobacter vinelandii |  | Activator | Schramm V L, et al. Biochemistry. 1971,10:3411-3417 |
| 5 | AMP-activated protein kinase | | Rat | P80385 | Activator | Sanders M J, et al. Biochem J. 2007,403:139-148. |
| 6 | 5'-AMP-activated protein kinase subunit beta-2 | | Human | O43741 | Activator | Sanders M J, et al. Biochem J. 2007,403:139-148 |
| 7 | Aspartate carbamoyltransferase | | Sulfolobus acidocaldarius | Q55338 | Activator | Tsuruta H, et al. J Mol Biol. 2005,348:195-204 |
| 8 | Aspartate carbamoyltransferase | | E.coli | P0A786 | Activator | Tsuruta H, et al. J Mol Biol. 2005,348:195-204 |
| 9 | Aspartate carbamoyltransferase | | Sulfolobus acidocaldarius | P74766 | Activator | De Vos D, et al. Biochem Biophys Res Commun. 2008,372:40-44. |
| 10 | Sulfate adenylyltransferase | | Penicillium chrysogenum | Q12650 | Activator | MacRae I J, et al. Biochemistry. 2001,40:6795-6804.; MacRae I J, et al. Nat.Struct.Biol. 2002,9:945-949. |
| 11 | Sulfate adenylyltransferase | | E.coli | P23845 | Activator | Wang R, et al. Biochemistry. 1995,34:490-495 |
| 12 | Cytochrome c oxidase | | Bovine | P00396 | Inhibitor | Nicholls P, et al. Biochim Biophys Acta. 1975,396:24-35 |
| 13 | Cytosolic purine 5'-nucleotidase | | Human | P49902 | Inhibitor | Wallden K, et al. J Biol Chem. 2007,282:17828-17836. |
| 14 | Fructose-bisphosphate aldolase | | Human | P04075 | Inhibitor | Sygusch J, et al. Biochem.J. 1997,327:717-720. |
| 15 | Fructose-bisphosphate aldolase | | Rabbit | P00883 | Inhibitor | Sygusch J, et al. Biochem.J. 1997,327:717-720. |
| 16 | Fructose-bisphosphate aldolase | | Fruit fly | P07764 | Inhibitor | Lal A, et al. Phytochemistry. 2005,66:968-974 |
| 17 | Glutamate dehydrogenase 1, mitochondrial | | Bovine | P00366 | Inhibitor | Smith TJ, et al. Trends.Biochem.Sci. 2008,33:557-564. |
| 18 | Glutamate dehydrogenase 1, mitochondrial | | Human | P00367 | Inhibitor | Smith TJ, et al. Trends.Biochem.Sci. 2008,33:557-564. |
| 19 | Glutamate dehydrogenase 1, mitochondrial | | Rat | P10860 | Inhibitor | Pazhanisamy S, et al. Biochemistry. 1994,33:10381-10385 |
| 20 | Glycogen phosphorylase | | Human | P11217 | Inhibitor | Lukacs CM, et al. Proteins. 2006,63:1123-1126. |
| 21 | Glycogen phosphorylase | | Rabbit | P06737 | Inhibitor | Rath VL, et al. Mol.Cell. 2000,6:139-148. |
| 22 | Glycogen phosphorylase | | Baker's yeast | P00489 | Inhibitor | Oikonomakos NG, et al. J Biol Chem. 2000,275:34566-34573.;Johnson LN, et al. J Mol Biol. 1993,232:253-267.; Oikonomakos NG, et al. Biochim Biophys Acta. 2003,1648:325-332.; Kurganov BI, et al. Biochemistry. 2000,39:13144-13152.; Tsitsanou KE, et al. Arch Biochem Biophys. 200,384:245-254. |
| 23 | Glycogen synthase | | Human | P06738 | Inhibitor | Bresler S, et al. Nature. 1996,211:1262-1263 |
| 24 | Glycogen synthase | | Rat | P13807 | Inhibitor | Rothman L B, et al. Biochemistry. 1967,6:2098-2106 |
| 25 | Glycogen synthase | | Baker's yeast | A2RRU1 | Inhibitor | Rothman L B, et al. Biochemistry. 1967,6:2098-2106 |
| 26 | 60 kDa chaperonin | | E.coli | P23337 | Inhibitor | Rothman L B, et al. Biochemistry. 1967,6:2098-2106 |
| 27 | Chaperone protein dnaK | | Thermus thermophilus | P0A6F5 | Activator | Roseman AM, et al. Cell. 1996,87:241-251.; Inobe T, et al. J.Mol.Biol. 2004,339:199-205. |
| 28 | Chaperone protein dnaK | | E.coli | Q56235 | Regulator | Revington M, et al. J.Mol.Biol. 2005,349:163-183. |
| 29 | DNA repair protein RAD51 homolog 1 | | Human | P0A6Y8 | Regulator | Swain JF, et al. Mol.Cell. 2007,26:27-39. |
| 30 | Insulin-degrading enzyme | | Rat | Q06609 | Activator | Renodon-Corniere A, et al. J.Mol.Biol. 2008,383:575-587. |
| 31 | Insulin-degrading enzyme | | Human | P35559 | Activator | Grasso G, et al. Biochim.Biophys.Acta. 2008,1784:1122-1126. |
| 32 | NAD-dependent malic enzyme | | Human | P14735 | Activator | Song ES, et al. J.Biol.Chem. 2005,280:17701-17706 |
| 33 | NAD-dependent malic enzyme | | Streptococcus faecalis | P23368 | Inhibitor | Su KL, et al. Bioorg.Med.Chem. 2009,17:5414-5419. |
| 34 | NAD-dependent malic enzyme | | Ascaris lumbricoides |  | Inhibitor | Su KL, et al. Bioorg.Med.Chem. 2009,17:5414-5419 |
| 35 | NAD-dependent malic enzyme | | Neurospora crassa | P27443 | Inhibitor | Su KL, et al. Bioorg.Med.Chem. 2009,17:5414-5419 |
| 36 | Mitochondrial brown fat uncoupling protein 1 | | Golden hamster |  | Inhibitor | Su KL, et al. Bioorg.Med.Chem. 2009,17:5414-5419 |
| 37 | Myosin V | | Chicken | P04575 | Regulator | Jezek P, et al. FEBS.Lett. 1995,361:303-307 |
| 38 | 6-phosphofructokinase isozyme 2 | | E.coli | Q02440 | Inhibitor | Tehver R, et al. Structure. 2010,18:471-481.; Jackson DR Jr, et al. Phys.Chem.Chem.Phys. 2009,11:4808-4814. |
| 39 | Pyruvate kinase | | Baker's yeast | P06999 | Inhibitor | Guixe V, Biochemistry. 1998,37:13269-13275. |
| 40 | Pyruvate kinase | | Human | P00549 | Inhibitor | Wieker HJ, et al. Biochemistry. 1971,10:1243-1248.; Bond CJ, Biochemistry. 2000,39:15333-15343. |
| 41 | Pyruvate kinase | | Rabbit | P14618 | Inhibitor | Dombrauckas JD, et al. Biochemistry. 2005,44:9417-9429. |
| 42 | Pyruvate kinase | | E.coli | P30613 | Inhibitor | Valentini G, et al. J.Biol.Chem. 2002,277:23807-23814. |
| 43 | Pyruvate kinase | | Rat | P11974 | Inhibitor | Williams R, et al. Biochemistry. 2006,45:5421-5429.;  Herman P, et al. Biochemistry. 2009,48:9448-9455. |
| 44 | Pyruvate kinase | | Leishmania mexicana | P0AD61 | Inhibitor | Mattevi A, et al. Structure. 1995,3:729-741. |
| 45 | Protein recA | | E.coli | P11980 | Inhibitor | Abraham DJ, et al. US 6534501 |
| 46 | Protein recA | | Mycobacterium smegmatis | P12928 | Inhibitor | Abraham DJ, et al. US 6534501 |
| 47 | Ribonucleotide reductase | | E.coli | Q27686 | Inhibitor | Abraham DJ, et al. US 6534501 |
| 48 | Ribonucleotide reductase | | Thermotoga maritima | P0A7G6 | Activator | Kelley De Zutter J, et al. Structure. 2001,9:47-55.;  Voloshin ON, et al. J.Mol.Biol. 2000,303:709-720. |
| 49 | Ribonucleotide reductase | | Salmonella typhimurium | Q59560 | Activator | Kelley De Zutter J, et al. Structure. 2001,9:47-55. |
| 50 | Ribonucleotide reductase | | Lactobacillus leichmannii | P00452 | Regulator | Eriksson M, et al. Structure. 1997,5:1077-1092. |
| 51 | ATP-dependent DNA helicase RuvB | | E.coli |  | Regulator | Larsson KM, et al. Nat.Struct.Mol.Biol. 2004,11:1142-1149. |
| 52 | Sarcoplasmic/endoplasmic reticulum calcium ATPase | | Rabbit | Q08698 | Regulator | Uppsten M, et al. J.Mol.Biol. 2003,330:87-97. |
| 53 | Serine Racemase | | Mouse | Q59490 | Regulator | Sintchak MD, et al. Nat.Struct.Biol. 2002,9:293-300. |
| 54 | UDP-glucuronosyltransferase 1A1 | | Rat | P0A812 | Inhibitor | Hishida T, et al. Proc.Natl.Acad.Sci U S A. 2004,101:9573-9577. |
| 55 | UDP-glucuronosyltransferase 1A1 | | Human | P04191 | Activator | Singh P, et al. J.Med.Chem. 2005,48:3005-3014.;  Zamoon J, et al. Proc.Natl.Acad.Sci U S A. 2005,102:4747-4752. |
| 56 | UMP kinase | | Bacillus anthracis | P20647 | Activator | Singh P, et al. J.Med.Chem. 2005,48:3005-3014 |
| 57 | UMP kinase | | E.coli | Q9QZX7 | Activator | Neidle A, et al. Neurochem.Res. 2002,27:1719-1724. |
| 58 | Uridylate kinase | | Xanthomonas campestris pv. campestris | Q64550 | Inhibitor | Nishimura Y, et al. Biochim.Biophys.Acta. 2007,1770:1557-1566 |
| 59 | Uridylate kinase | | Mycobacterium tuberculosis | P22309 | Inhibitor | Nishimura Y, et al. Biochim.Biophys.Acta. 2007,1770:1557-1566 |
| 60 | Alpha 1,4-glucan phosphorylase | | Banana |  | Activator | Meier C, et al. J.Mol.Biol. 2008,381:1098-1105. |
| 61 | 6-phosphofructokinase | | Cynomolgus monkey | P0A7E9 | Activator | Marco-Marin C, et al. FEBS Lett. 2009,583:185-189. |
| 62 | 6-phosphofructokinase | | Rabbit | P59009 | Activator | Meier C, et al. J.Mol.Biol. 2008,381:1098-1105 |
| 63 | 6-phosphofructokinase | | E.coli | P65929 | Activator | Meier C, et al. J.Mol.Biol. 2008,381:1098-1105 |
| 64 | 6-phosphofructokinase | | Geobacillus stearothermophilus |  | Inhibitor | Singh S, et al. Biochim.Biophys.Acta. 1973,309:280-288 |
| 65 | Primosomal protein N' | | E.coli | Q60HD9 | Inhibitor | Kondo S, et al. J Invest Dermatol. 1972,59:397-401 |
| 66 | Hemoglobin | | Human | P00511 | Inhibitor | Kondo S, et al. J Invest Dermatol. 1972,59:397-401 |
| 67 | ATP-dependent Clp protease ATP-binding subunit clpX | | E.coli | P0A796 | Inhibitor | Shirakihara Y, et al. J Mol Biol. 1988,204:973-994. |
| 68 | Atrial natriuretic peptide receptor 1 | | Human | P00512 | Inhibitor | Kondo S, et al. J Invest Dermatol. 1972,59:397-401 |
| 69 | DNA mismatch repair protein mutS | | E.coli | P17888 | Regulator | Lucius A L, et al. Biochemistry. 2006,45:7237-7355. |
| 70 | Myosin-2 heavy chain | | Slime mold | P69905 | Regulator | Yokoyama T, et al. J Mol Biol. 2006,356:790-801.;  Boyiri T, et al. Biochemistry. 1995,34:15021-15036.;  Abraham DJ, et al. Biochemistry. 1992,31:9141-9149. |
| 71 | Glutamate dehydrogenase 2, mitochondrial | | Human | P68871 | Regulator | Abraham DJ, et al. Biochemistry. 1992,31:9141-9149.;  Yokoyama T, et al. J Mol Biol. 2006,356:790-801. |
| 72 | Polyribonucleotide nucleotidyltransferase | | E.coli | P0A6H1 | Activator | Hersch GL, et al. Cell. 2005,121:1017-1027.;  Glynn SE, et al. Cell. 2009,139:744-756 |
| 73 | DNA topoisomerase 2 | | Baker's yeast | P16066 | Activator | Duda T, et al. Biochemistry. 2011,50:1213-1225. |
| 74 | Transcriptional regulatory protein xylR | | Pseudomonas putida | P23909 | Activator | Lamers MH, et al. J Biol Chem. 2004,279:43879-43885. |
| 75 | Chromosomal replication initiator protein DnaA | | E.coli | P08799 | Regulator | Vileno B, et al. Proc Natl Acad Sci U S A. 2011,108:8218-8223. |
| 76 | Lon protease | | E.coli | P49448 | Inhibitor | Plaitakis A, et al. J Neurochem. 2000;75:1862-1869 |
| 77 | UDP-glucuronosyltransferase 1-9 | | Human | P05055 | Inhibitor | Nurmohamed S, et al. J Biol Chem. 2011;286:14315-14323 |
| 78 | P2X4 receptor | | Zebrafish |  | Activator | Hattori M, et al. Nature. 2012, 485,207-213. |
