# Supplementary material for: The Structural Basis of ATP as an Allosteric Modulator
Source: PLoS Comput Biol. 2014 Sep 11;10(9):e1003831. doi: 10.1371/journal.pcbi.1003831 (PMC4161293; doi:10.1371/journal.pcbi.1003831)
Supplement: Table S7 — Summary of MD simulation systems. (DOC) [file pcbi.1003831.s012.doc]

**Table S7:** Summary of MD simulation systems

| *System name* | *Simulation method* | *Simulation time (ns)* |
| --- | --- | --- |
| ATP in solution | Unbiased | 2000 |
| Unbound UMP kinase | Unbiased | 100 |
| Bound UMP kinase | Unbiased | 100 |
| ATP access to allosteric site of UMP kinase | NEB | 0.9×20 |
| ATP access to substrate site of UMP kinase | NEB | 0.9×20 |
| Total |  | 2236 |
