# Supplementary material for: The Structural Basis of ATP as an Allosteric Modulator
Source: PLoS Comput Biol. 2014 Sep 11;10(9):e1003831. doi: 10.1371/journal.pcbi.1003831 (PMC4161293; doi:10.1371/journal.pcbi.1003831)
Supplement: Table S8 — Simulated annealing protocol used for minimization in NEB simulations. (DOC) [file pcbi.1003831.s013.doc]

**Table S8:** Simulated annealing protocol used for minimization in NEB calculations

| *Step* | *Step*  *numbers* | *NEB spring*  *force constant* | *Procedure*  *type* | *Start*  *temperature*  *(K)* | *End*  *temperature*  *(K)* |
| --- | --- | --- | --- | --- | --- |
| 1 | 20000 | 50 | NVT MD  with heat up | 300 | 500 |
| 2 | 10000 | 50 | NVT MD  first cooling | 500 | 400 |
| 3 | 10000 | 50 | NVT MD  second cooling | 400 | 300 |
| 4 | 50000 | 50 | NVT MD  room temperature | 300 | 300 |
| 5 | 10000 | 50 | NVT MD  slow cooling | 300 | 250 |
| 6 | 2000 | 50 | NVT MD equilibrium | 250 | 250 |
| 7 | 10000 | 50 | NVT MD  slow cooling | 250 | 200 |
| 8 | 2000 | 50 | NVT MD equilibrium | 200 | 200 |
| 9 | 10000 | 50 | NVT MD  slow cooling | 200 | 150 |
| 10 | 2000 | 50 | NVT MD equilibrium | 150 | 150 |
| 11 | 10000 | 50 | NVT MD  slow cooling | 150 | 100 |
| 12 | 2000 | 50 | NVT MD equilibrium | 100 | 100 |
| 13 | 10000 | 50 | NVT MD  slow cooling | 100 | 50 |
| 14 | 2000 | 50 | NVT MD equilibrium | 50 | 50 |
| 15 | 50000 | 50 | NVT MD  slow cooling | 50 | 0 |
| 16 | 100000 | 50 | Quenched MD | 0 | 0 |
